# Pre-clinical efficacy of a candidate outer membrane vesicle gonococcal vaccine in comparison with 4CMenB

**DOI:** 10.1038/s41541-026-01491-z

**Published:** 2026-06-08

**Authors:** Paula Freixeiro, Lisa A. Lewis, Leonor Sánchez-Busó, Magnus Unemo, Sunita Gulati, Aline Linder, Jukka Corander, Odile B. Harrison, Christina Dold, Simon Harris, Christine S. Rollier, Sanjay Ram, Calman A. MacLennan

**Affiliations:** 1 Jenner Institute, Nuffield Department of Medicine, University of Oxford, Oxford, UK; 2 University of Massachusetts, Worcester, MA USA; 3 Center for Scientific Review, National Institutes of Health, Bethesda, MD USA; 4 Joint Research Unit ‘Infection and Public Health, Institute for Integrative Systems Biology (I2SysBio), FISABIO-University of Valencia, Valencia, Spain; 5 Centro de Investigación Biomédica en Red de Epidemiología y Salud Pública, ISCIII, Madrid, Spain; 6 WHO Collaborating Centre for Gonorrhoea and Other STIs, Örebro University, Örebro, Sweden; 7 Institute for Global Health, University College London, London, UK; 8 Oxford Vaccine Group, Department of Paediatrics, University of Oxford, Oxford, UK; 9 Institute of Basic Medical Sciences, University of Oslo, Oslo, Norway; 10 Nuffield Department of Population Health, University of Oxford, Oxford, UK; 11 Wellcome Sanger Institute, Wellcome Genome Campus, Hinxton, UK; 12 Microbiotica, Cambridge, UK; 13 School of Biosciences, University of Surrey, Guildford, UK; 14 Department of Immunology and Immunotherapy, University of Birmingham, Birmingham, UK

**Keywords:** Diseases, Immunology, Microbiology

## Abstract

*Neisseria gonorrhoeae* causes 82 million global cases of gonorrhoea annually. Multidrug-resistant gonococci threaten to make gonorrhoea untreatable. Meningococcal vaccines MeNZB and 4CMenB (Bexsero), containing *Neisseria meningitidis* group B detergent-extracted outer membrane vesicles (dOMV), cross-protect against gonorrhoea with 31–59% effectiveness. We hypothesised that gonococcal OMV-based vaccines would have greater efficacy against gonorrhoea than meningococcal vaccines. We developed native OMV (nOMV) candidate *Neisseria* vaccines from gonococcal strains GC_0817560 and FA1090, and meningococcal B strain NZ98/254. *lpxL1* and *rmp* genes were deleted to reduce reactogenicity and minimise induction of unprotective or blocking antibodies. nOMV were characterised and formulated with aluminium hydroxide. Deletion of *lpxL1* markedly reduced nOMV-induced IL-6 release from peripheral blood mononuclear cells. nOMV derived from GC_0817560*lpxL1*^*−*^*rmp*^*−*^ and FA1090*lpxL1*^*−*^*rmp*^*−*^ induced greater quantities of gonococcal-specific serum IgG and accelerated clearance of FA1090 from oestradiol-treated BALB/c mice significantly faster than 4CMenB and NZ98/254*lpxL1*^*−*^*rmp*^*−*^ nOMV (*P* < 0.0001). Gonococcal nOMV-based vaccines represent promising candidates for further development.

## Introduction

*Neisseria gonorrhoeae* (gonococcus) is an obligate human pathogen and the aetiological agent of gonorrhoea, the second most common of the reported bacterial sexually transmitted infections (STIs)^1^. There are an estimated 82 million new global cases among adults (15–49 years of age) each year^1^ with marginalised groups and those in low- and middle-income countries, particularly in sub-Saharan Africa, disproportionately affected^2^. Gonorrhoea is most commonly acquired through sexual contact and establishes infection in the urogenital tract, causing urethritis in men and cervicitis in women. Rectal and pharyngeal gonococcal infections are also common. Urogenital infection can result in severe complications and sequelae, including pelvic inflammatory disease, infertility, ectopic pregnancy, neonatal conjunctivitis leading to blindness and disseminated gonococcal infection and facilitates the transmission and acquisition of other STIs, including HIV infection^3^.

Infection management is complicated by high numbers of asymptomatic cases and the ability of the gonococcus to develop resistance to all available antibiotics. The prevalence of multidrug-resistant gonococcal strains, with resistance to ceftriaxone, azithromycin or both, has substantially increased during the past decade^2,4–6^ and consequently the WHO has issued an alert that gonorrhoea may become untreatable^7^. Despite two promising antimicrobials which recently received US FDA approval for treatment of uncomplicated gonorrhoea, gepotidacin^8^ and zoliflodacin^9^, there is an urgent need for a vaccine against this pathogen.

Only three vaccine candidates, a killed whole cell vaccine^10^, purified pilin vaccine^11^ and purified gonococcal porin (PorB) vaccine^12^, have been tested in prospective clinical efficacy trials in humans, but none were efficacious. During the past few years, new immunogenic gonococcal outer membrane proteins, which are less polymorphic and more conserved than PorB, have been identified. These include MtrE, a component of the MtrCDE multidrug efflux pump; transferrin-binding proteins A and B (TbpA and TbpB); and lactoferrin-binding proteins A and B (LbpA and LbpB). Due to their higher level of antigenic conservation and stable expression, such antigens could serve as potential vaccine targets^13^. However, the high antigenic and phase variation of gonococcal antigens make a single antigen strategy unlikely to be successful, and, similar to the strategy used for vaccine development against *Neisseria meningitidis* serogroup B, a combination of different antigens is perhaps more likely to be successful. In 2017, a retrospective case-control study in young adults found that immunisation with MeNZB, an *N. meningitidis* serogroup B dOMV vaccine, was associated with moderate protection against gonorrhoea (estimated vaccine effectiveness = 31%, 95%CI 21, 39)^14^. MeNZB consists of detergent-extracted outer membrane vesicles (dOMV) from strain NZ98/254, and this dOMV is also a component of the GSK 4CMenB (Bexsero) meningococcal group B vaccine.

There are several obstacles to gonococcal vaccine development, including a lack of understanding of what immune response is required for protection, no identified correlates of protection^13,15^, high levels of antigenic variability and production of potentially blocking antibodies against conserved membrane antigens. Natural infection with the gonococcus typically induces an inflammatory response characterised by neutrophil influx but does not usually result in protective immunity^16^. Recurrent gonococcal infections are frequent, and antibody production, both local and systemic, is minimal and does not appear to be protective^17^.

Following the success of largely strain-specific dOMV vaccines against serogroup B meningococcus, OMV have been studied as vaccine candidates for several microorganisms^18,19^. As well as licenced vaccines against serogroup B *N. meningitidis*, OMV vaccines against *Shigella* and nontyphoidal *Salmonella* are being assessed in clinical trials^19,20^. OMV technology has diversified and now includes nOMV^19^, which exploit the capacity of Gram-negative bacteria to release outer membrane blebs containing outer membrane proteins and lipo-oligosaccharide or lipopolysaccharide while maintaining the native orientation and conformation of these antigens. The detergent-extraction used to release dOMV from the bacterial cytoplasm reduces lipo-oligosaccharide content and has unacceptable reactogenicity in humans. However, detergent extraction removes lipoproteins, which contribute to immunogenicity and protection^21^, resulting in changes to dOMV structure, and promotes aggregation^22^.

The use of nOMV avoids the need to employ detergents for vesicle purification, but the parental bacterial strain has to be modified to reduce reactogenicity of lipo-oligosaccharide and may also be altered to increase nOMV yield. In Gram-negative bacteria, lipid A is the endotoxic component of lipo-oligosaccharide and drives reactogenicity^23^. Deletion of lipid A biosynthesis genes (e.g. *lpxL1*) results in under-acylated lipid A, reducing reactogenicity while retaining adjuvant activity^24^. Reduction modifiable protein (Rmp, or PIII, encoded by *rmp*) induces the formation of antibodies that can potentially block the bactericidal activity of antibodies targeting other gonococcal antigens^25,26^. Although the structure and function of this protein are not fully understood, Rmp is involved in bacterial adhesion to human cells^27^ and meningococcal homologous protein RmpM is involved in the stabilisation of outer membrane protein complexes^28,29^. Deletion of meningococcal RmpM increases the release of nOMV by disrupting the integrity of the attachment of the inner and outer membranes^22^. Therefore, deletion of *rmp* could prevent the formation of blocking antibodies and increase the production of nOMV.

In this study, we design, produce and characterise gonococcal and meningococcal nOMV generated from bacteria with deleted *lpxL1* and *rmp* genes. We then compare the ability of these candidate vaccines to accelerate the clearance of the gonococcus in a mouse infection model in comparison to the group B meningococcal vaccine, 4CMenB. Our findings indicate that these nOMV are straightforward to produce, have low reactogenicity, and that gonococcal nOMV accelerate clearance of gonococcal infection in mice relative to 4CMenB and placebo.

## Results

### Selection of *Neisseria gonorrhoeae* strain GC_0817560

**Fig. 1:**
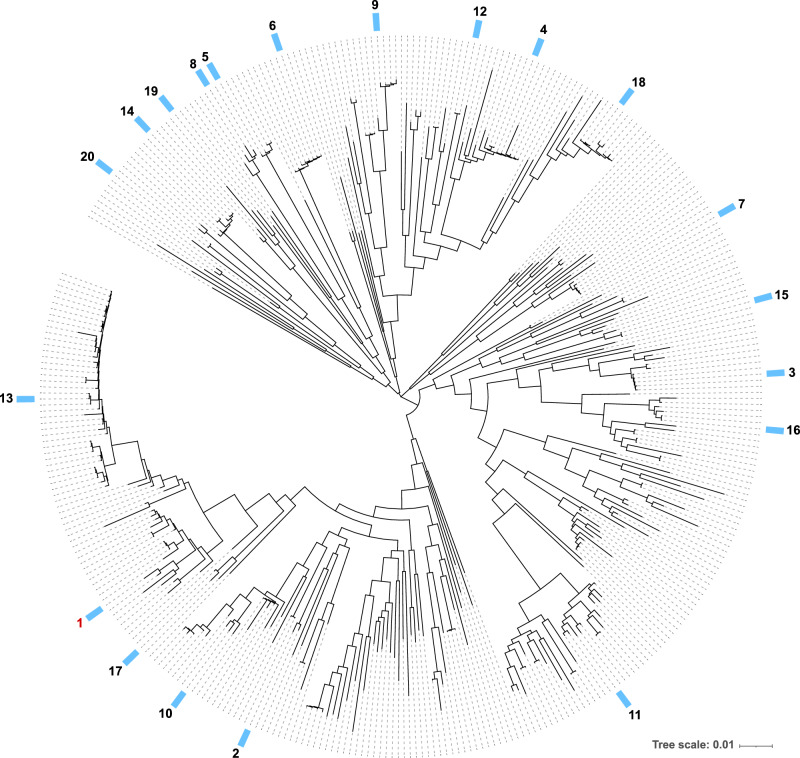
Phylogenetic tree of 362 global *Neisseria gonorrhoeae* isolates constructed from 57,310 polymorphic sites (SNPs) derived from whole genome sequence data. The 20 isolates representative of the diversity of five key antigens (*lbpA, lbpB, tbpA*, *tbpB* and *mtrE*) selected for nOMV production are indicated on the tree. The scale bar represents the expected number of nucleotide substitutions per variable site along each branch. Identities, characteristics and nOMV production of the 20 chosen isolates are in Table 1.

A panel of twenty gonococcal strains (Table 1) representative of the diversity of five potential vaccine antigens, LbpA, LbpB, TbpA, TbpB and MtrE, was selected from a collection of 362 global isolates from the WHO Collaborating Centre for Gonorrhoea and other Sexually Transmitted Infections, Örebro (Fig. 1 and Supplementary Fig. 1). The selection was performed by calculating pairwise strain distances as the sum of distances in each protein-based antigen phylogenetic tree, projecting these distances using MDS to define centroids and identifying the 20 strains that maximise representation of the diversity of the five antigens. Selected gonococcal isolates were screened as potential vaccine production strains by their ability to release nOMV following culture in shake flasks, with the total protein content of released nOMV used to estimate nOMV production yield.

**Table 1:** Characteristics of 20 *N. gonorrhoeae* strains assessed for ability to produce nOMV and challenge strain (FA1090)

	Strain	Accession number	Year of isolation	Country of isolation	MLST ST	NG-MAST			NG-STAR ST	nOMV yield (mg)
						ST	*porB*	*tbpB*		
1	GC_0817560	ERR349896	2008	Chile	1901	3795	2301	4	291	0.97
2	989000059	ERR349900	1998	USA	7367	270	206	33	6705	0.35
3	989000124	ERR349919	1998	Denmark	1579	21	14	33	139	0.173
4	989000378	ERR349924	1998	France	1584	85	70	24	178	0.37
5	989000439	ERR349927	1998	Germany	6726	19095	383	189	1526	0.34
6	03_590_96	ERR349964	2002	Denmark	8122	292	28	4	85	0.54
7	IN_10_151	ERR363662	2010	India	8776	6064	3609	33	285	0.65
8	590_302	ERR388301	2008	Turkey	11177	1993	1257	6	568	0.89
9	SI08_07	ERR388306	2008	Slovenia	8114	4	3	4	46	0.33
10	BEL_09_37	ERR388330	2009	Belarus	8126	1929	1223	29	182	0.15
11	SI09_11	ERR388333	2009	Slovenia	1588	5569	489	137	247	0.71
12	EST_10_22	ERR388340	2010	Estonia	1931	7487	26	131	269	0.51
13	10_590_246	ERR388352	2010	Spain	1901	1407	908	110	90	0.33
14	EST_11_24	ERR388372	2011	Estonia	1594	7482	4519	27	20	-
15	POL_11_148	ERR388393	2011	Poland	10,932	8392	5050	263	169	0.36
16	BX290	ERR449458	2011	Pakistan	1903	6320	3728	136	614	-
17	09_18	ERR449473	2011	Vietnam	7371	4787	1275	29	501	0.62
18	08_590_00133	ERR449507	2008	Guinea-Bissau	8112	3176	1923	60	353	0.62
19	08_590_00145	ERR449515	2008	Guinea-Bissau	1591	3188	123	60	357	-
20	02_231	ERR485168	2002	UK	1892	261	197	11	548	-
21	FA1090	NC_002946.2	1980	USA	1899	773	514	219	11	ND

nOMV yield was studied in 100 ml cultures and expressed as protein content measured by the Lowry assay. Strain GC_0817560, isolated in Chile in 2008, showed the highest nOMV yield and was selected for vaccine development. MLST ST multi-locus sequence typing sequence type. NG-MAST *Neisseria gonorrhoeae* Multi-Antigen Sequence Typing. NG-STAR *N. gonorrhoeae* Sequence Typing for Antimicrobial resistance. ND Not done.

Strain GC_0817560, isolated from a patient infected in Chile in 2008, gave the greatest nOMV yield (Table 1) and was chosen as the parental strain for nOMV candidate vaccine production. The common gonococcal laboratory strain, FA1090, which is used as the challenge strain in the gonococcal mouse infection model, was also used as a gonococcal nOMV production strain. The meningococcal NZ98/254 strain (dOMV extracted from NZ98/254 are a component of 4CMenB) was used as the meningococcal nOMV production strain.

At the genome-wide level, *N. gonorrhoeae* GC_0817560 and FA1090 strains show 99.7% identity as calculated by cgMLST through Pathogenwatch (3,044 SNPs in 1,470,119 nucleotides spanning 1542 gene sequences)^30^. These strains showed a very high identity in the MtrCDE outer membrane subunit MtrE, both at the nucleotide and protein levels (>99%). Protein level identity was above 96.4% for the transferrin-binding protein A (TbpA) but markedly lower for TbpB (82.6%) (Supplementary Table 1). FA1090 showed a disrupted lactoferrin-binding protein A (*lbpA*) gene and lacked a *lbpB* gene. When GC_0817560 was compared with *N. meningitidis* NZ98/254, protein identities were nearly 97% for MtrE and above 90% for TbpA and the lactoferrin-binding protein A (LbpA). However, protein identity was below 70% for TbpB and LbpB (Supplementary Table 2). The outer membrane protein PorB was also compared among the three strains, showing a protein identity of 93.4% between *N. gonorrhoeae* GC_0817560 and FA1090 and an identity of 67.4% between GC_0817560 and the *N. meningitidis* NZ98/254 strain.

**Fig. 2:**
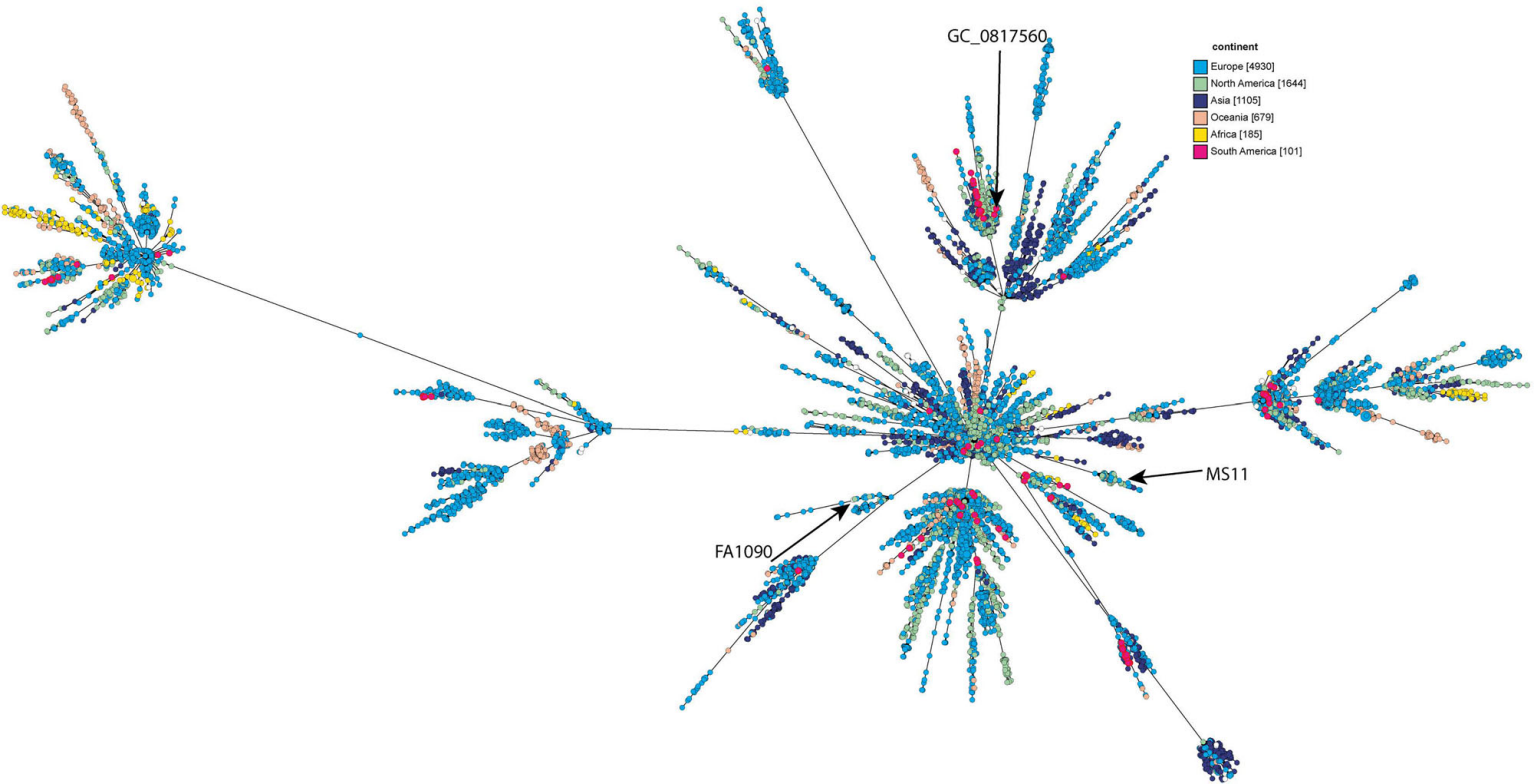
Minimum spanning tree comparing core genome allelic profiles, cgMLST V1.0, from 8679 gonococcal genomes stored in PubMLST^52,60^. Each node represents one isolate, and isolates with similar allelic profiles in the core genome form clusters. Nodes are coloured by continent with the commonly used reference strains FA1090 and MS11 indicated, as well as the Chilean strain, GC_0817560. Analyses indicate limited geographical associations between core genome clusters.

GC_0817560 belongs to core genome group 3, a large collection of isolates, predominantly ST1901, that circulate globally. Core genome comparison using cgMLST V1.0 revealed limited global associations with isolates clustering with GC_0817560 originating from all continents examined (Fig. 2).

### Genetic manipulation

To reduce nOMV reactogenicity, the *lpxL1* genes of nOMV production strains were replaced with a kanamycin cassette. To prevent the induction of non-protective or blocking antibodies and increase nOMV yield, the *rmp* genes were replaced with a chloramphenicol cassette. Deletions were confirmed by Sanger sequencing. Genetically modified strains showed good growth, although slightly slower than the wild type strains, and *lpxL1*^*−*^*rmp*^*−*^ strains reached a slightly lower final OD_600_ (Supplementary Fig. 2).

### Production of nOMV

**Fig. 3:**
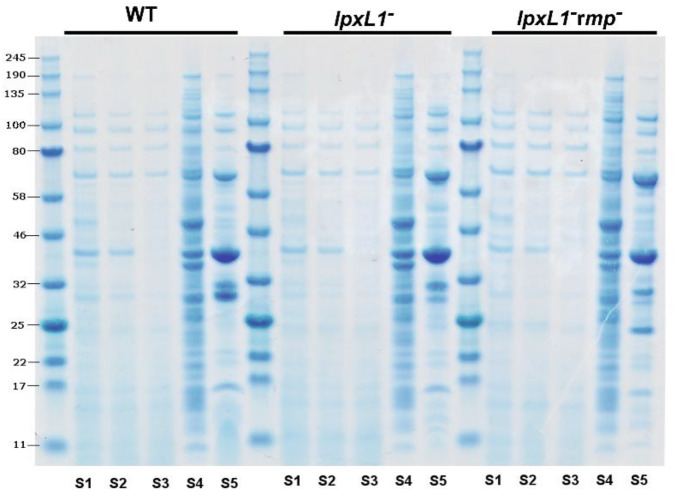
SDS-PAGE analysis of nOMV from GC_0817560 wild-type (WT) and genetically modified strains. From the original culture material, cells were removed by centrifugation, and the supernatants containing nOMV and soluble proteins were filtered to remove cellular debris, followed by ultracentrifugation to remove soluble proteins. Pellets containing nOMV were resuspended in PBS and filtered to obtain the nOMV candidate vaccines. Samples were collected during production of nOMV: S1 whole culture, S2 supernatant containing nOMVs and soluble proteins, S3 soluble proteins fraction, S4 cells fraction, S5 concentrated nOMVs.

**Fig. 4:**
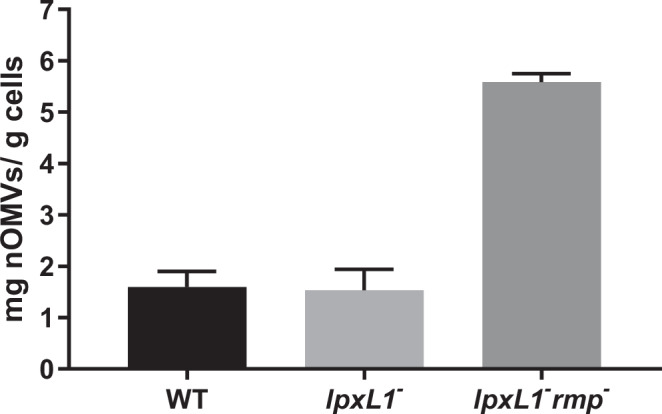
Production of nOMV from GC_0817560 wild-type (WT) and genetically modified strains. Production yield of nOMV from the WT, *lpxL1*^*−*^ and *lpxL1*^*−*^*rmp*^*−*^ strains expressed as mg nOMV from g cells total culture biomass.

nOMV were purified from overnight bacterial culture supernatants using the centrifugation-filtration method described. Results of gonococcal nOMV production from wild-type, *lpxL1*^*−*^ *and lpxL1*^*−*^*rmp*^*−*^ GC_0817560 are shown as a production process model, and to show differences between the wild type and genetically modified strains. Samples were taken at different steps of the nOMV production process and analysed by SDS-PAGE (Fig. 3). Following low-speed centrifugation, supernatants were filtered to eliminate cell debris and ultracentrifuged to precipitate nOMV and eliminate soluble proteins and cellular debris, prior to resuspension in PBS and sterile filtration (Fig. 3). Differences in the protein pattern between cells and nOMV are apparent with a visible enrichment of several protein bands seen for nOMV. Genetically modified strains with deleted *rmp* had higher nOMV release than strains with intact *rmp* (Fig. 4). The *lpxL1*^*−*^*rmp*^*−*^ nOMV-production strains gave a 3.6-, 2.4- and 1.6-fold higher nOMV yield compared with wild type GC_0817560, FA1090 and NZ98/254 strains, respectively. nOMV produced from the different gonococcal strains screened to select a vaccine production strain showed similar patterns of proteins (Supplementary Fig. 3).

### Characterisation of nOMV

**Fig. 5:**
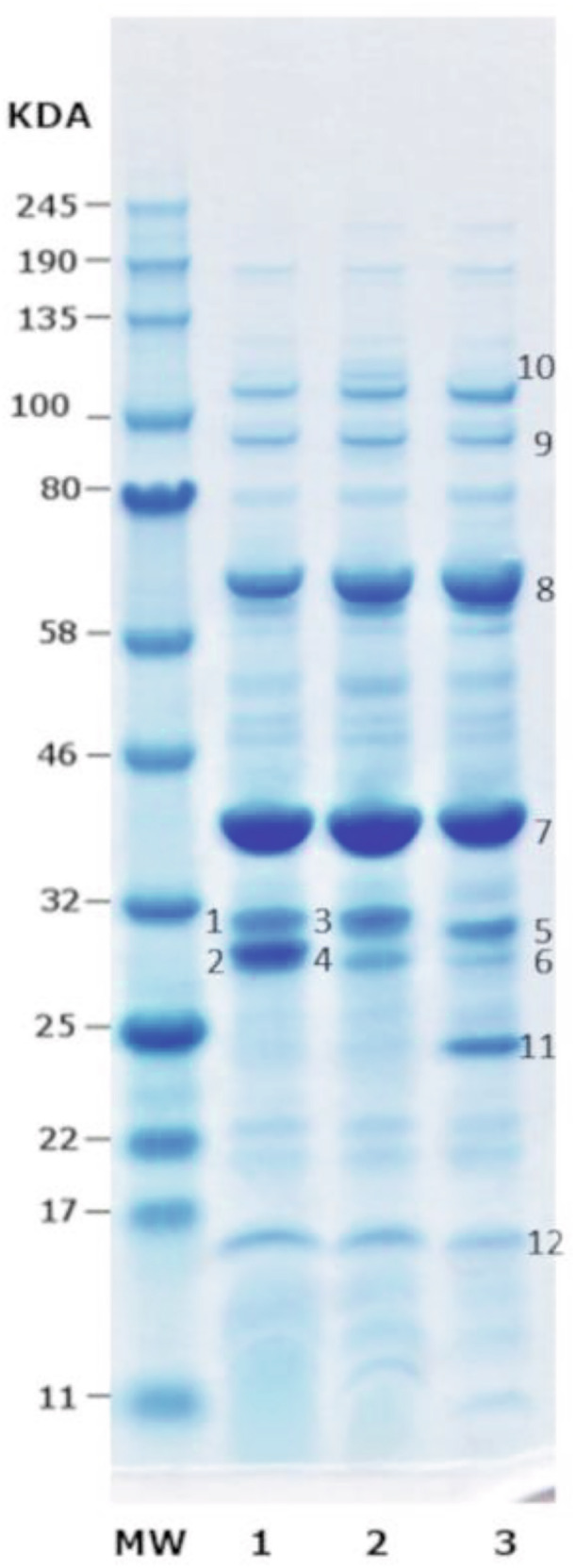
SDS-PAGE analysis of *N. gonorrhoeae* GC_0817560 nOMV. From wild type GC_0817560 (lane 1), GC_0817560 *lpxL1*^−^ (lane 2) and GC_0817560 *lpxL1*^*−*^*rmp*^*−*^ (lane 3), from which indicated protein bands were excised and identified by mass spectrometry. Eight micrograms of nOMV was loaded into each lane of a 4–12% polyacrylamide gradient gel using MES buffer. Gel was stained with Coomassie Blue.

The protein patterns obtained by electrophoretic analysis of nOMV from wild type, and *lpxL1*^*−*^*rmp*^*−*^ vaccine strains were compared (Supplementary Fig. 4), and the protein content of GC_0817560 nOMV was studied by SDS-PAGE with mass spectrometric identification of the main protein bands (Fig. 5 and Table 2). Rmp was identified in band 1 and 3 of wild-type and *lpxL1*^*−*^ forms of GC_0817560 nOMV, respectively, but it was not identified in the same region of the gel for GC_0817560*lpxL1*^*−*^*rmp*^*−*^ nOMV indicating the absence of this protein in the GC_0817560 strain with deleted *rmp*. The visually most abundant proteins in *lpxL1*^*−*^*rmp*^*−*^ nOMV were identified as outer membrane proteins, including PorB, chaperonin, opacity (Opa) proteins, components of the Bam system and TbpA, consistent with the concept that nOMV are enriched in outer membrane proteins.

**Table 2:** GC_0817560 OMV protein identification by mass spectrometry

Band	Protein identification	Mass (kDa)	Mascot Score
1	Rmp (Outer membrane protein P.III)	25.54	869
2	Opacity protein opA52	28.12	374
3	Rmp (Outer membrane protein P.III)	25.54	907
4	Outer membrane protein assembly factor BamD	30.84	684
5	Opacity protein V28	26.77	353
6	Outer membrane protein assembly factor BamD	30.84	631
7	Major outer membrane protein P.IB (PorB)	37.42	945
8	60 kDa chaperonin	57.24	2425
9	Outer membrane protein assembly factor BamA	87.94	782
10	Transferrin-binding protein 1 (TbpA)	101.58	1019
11	Opacity protein Opa60	27.07	297
12	ATP synthase subunit b	17.14	145

Proteins in nOMV were separated by SDS-PAGE as shown in Fig. 3, and excised bands were in-gel digested with trypsin and identified by LC-MS using a Bruker Esquire HTC ESI-ion trap mass spectrometer fitted with a standard ion source in positive ion mode.

**Fig. 6:**
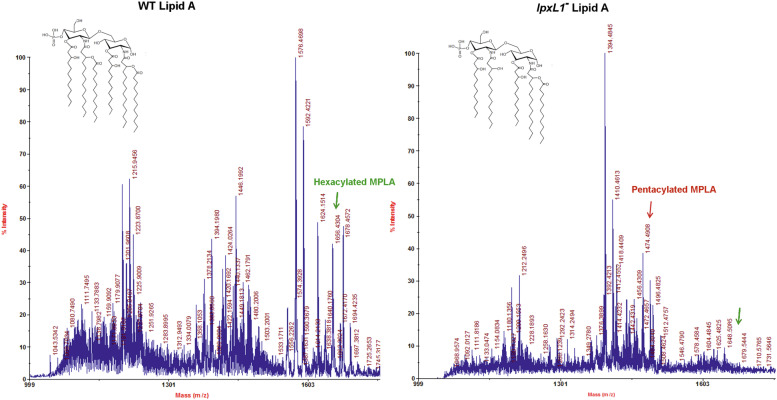
MALDI TOF/TOF analysis of purified GC_0817560 lipid A. Wild type (WT) and *lpxL1*^*−*^ lipo-oligosaccharide were purified by mild acid hydrolysis, and data were acquired in positive reflectron mode (*m/z* 800–2000) using a 4800plus MALDI-TOF/TOF (AB Sciex). Peaks corresponding to hexacylated (*m/z* 1656, WT strain) and pentacylated (*m/z* 1474, *lpxL1*^*−*^ strain) monophosphoryl lipid A (MPLA) are indicated.

To study the structural effect of *lpxL1* deletion, lipid A of GC_0817560 wild-type and *lpxL1*^*−*^ strains were analysed by MALDI-TOF/TOF. *lpxL1*^*−*^ monophosphoryl lipid A (MPLA) has an *m/z* shift corresponding to a theoretical mass of 182 Da compared to the wild type hexacylated MPLA (*m/z* 1656), resulting in a peak of *m/z* 1474, which is consistent with the loss of a lauroyl fatty acid group to give pentacylated lipid A (Fig. 6). No peak was present in the spectra of *lpxL1*^*−*^ lipid A at the corresponding mass for the hexacylated MPLA (*m/z* 1656). Equivalent peaks with lost phosphoryl groups (PO_4_^2−^, theoretical mass 80 Da) are also present in both spectra (*m/z* 1394 *lpxL1*^*−*^ and *m/z* 1576 WT lipid A).

**Fig. 7:**
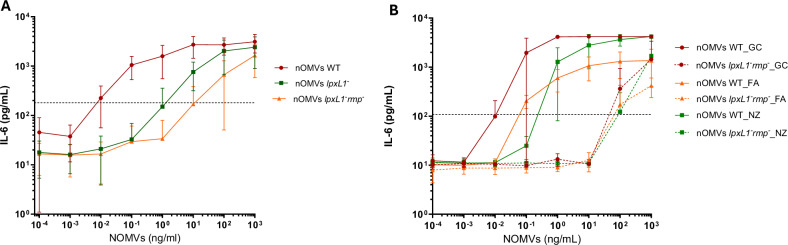
IL-6 production by human PBMCs in monocyte activation test (MAT) after stimulation with *Neisseria* nOMV. **A** Comparison of WT, *lpxL1*^*−*^ and *lpxL1*^*−*^*rmp*^*−*^ GC_0817560 nOMV. **B** Comparison of gonococcal strains GC_0817560 (GC) and FA1060 (FA) and meningococcal NZ98/254 (NZ) WT and *lpxL1*^*-*^*rmp*^*-*^ nOMV. PBMCs from three donors were stimulated with 0.0001–1000 ng/ml of WT, *lpxL1*^*−*^ or *lpxL1*^*−*^*rmp*^*−*^ nOMV. IL-6 production was measured in the supernatant by ELISA. Recombinant human IL-6 was used as standard and cytokine release by PBMCs exposed to PBS was used to determine background. IL-6 concentrations representing a 10-fold increase over background are indicated by a dashed horizontal line.

To confirm the reduced reactogenicity of the modified lipid A in the candidate vaccine nOMV, different concentrations of nOMV purified from the wild-type, *lpxL1*^*−*^ and *lpxL1*^*−*^*rmp*^*−*^ GC_0817560, FA1090 and NZ98/254 strains were used to stimulate human PBMCs in the MAT with release of the proinflammatory cytokine IL-6 measured by ELISA. nOMV purified from wild-type GC_0817560 with hexacylated lipid A induced the release of high levels of IL-6 while stimulation with nOMV with modified pentacylated lipid A resulted in substantially lower IL-6 release (Fig. 7). The amount of nOMV required to give a 10-fold increase in IL-6 release over background was approximately 100-fold and 1000-fold higher for nOMV from GC_0817560*lpxL1*^*−*^ and GC_0817560*lpxL1*^*−*^*rmp*^*−*^ respectively, compared with nOMV from the wild-type GC_0817560 (Fig. 7A and Table 3) indicating lower reactogenicity of the modified pentacylated lipid A. Wild-type nOMV from GC_0817560 induced higher IL-6 release than nOMV from FA1090 and NZ98/254, but nOMV from all three *lpxL1*^*−*^*rmp*^*−*^ genetically modified strains induced similar IL-6 levels (Fig. 7B), confirming their lower reactogenicity.

**Table 3:** Concentrations of nOMV derived from wild type, *lpxL1*^*−*^ and *lpxL1*^*−*^*rmp*^*−*^ GC_0817560 resulting in a 10-fold increase of IL-6 release over background in monocyte activation test (MAT)

nOMV concentrations (ng/ml)				
	Donor 1	Donor 2	Donor 3	Mean
**WT nOMV**	0.008	0.008	0.023	0.01
***lpxL1***^***−***^ **nOMV**	3.47	0.842	4.14	1.57
***lpxL1***^***−***^***rmp***^***−***^ **nOMV**	70.3	7.93	64.4	16.1

PBMCs cells from three donors were stimulated with 0.0001–1000 ng/ml of each form of nOMV and the release of IL-6 was measured by ELISA. Background IL-6 release was measured in cells incubated with PBS alone.

**Fig. 8:**
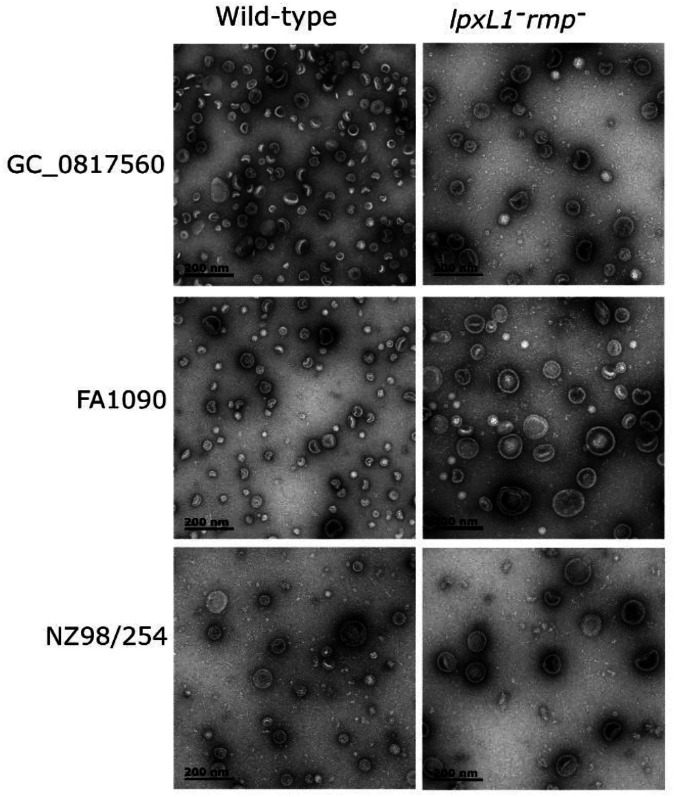
Transmission electron microscopy of purified wild-type and *lpxL*^*−*^*rmp*^*−*^ nOMV from GC_0817560, FA1090 and NZ98/254. Samples were diluted in PBS, negatively stained with 2% uranyl acetate and viewed with a FEI Tecnai T12 Transmission Electron Microscope. Size bars represent 200 nm.

nOMV from different wild-type and *lpxL1*^*−*^*rmp*^*−*^ *Neisseria* strains exhibited similar morphology by transmission electron microscopy (Fig. 8), characterised by spherical vesicles with preserved membrane integrity. Dynamic light scattering (DLS) analysis revealed that all nOMV present monodisperse populations (indicated by low polydispersity index (PDI)). Vesicles from the wild-type strains displayed a smaller average diameter compared to corresponding *lpxL1*^*−*^*rmp*^*−*^ strains. Additionally, nOMV from the two gonococcal strains had comparable sizes, which were significantly smaller than meningococcal nOMV (Table 4).

**Table 4:** Size distribution of *Neisseria* nOMV analysed by dynamic light scattering

Strain	nOMV	Diameter (nm) ± SD	PDI ± SD
**GC_0817560**	wild type	67.66 ± 2.88	0.216 ± 0.030
	*lpxL1*^*−*^*rmp*^*−*^	93.83 ± 5.29	0.245 ± 0.056
**FA1090**	wild type	76.62 ± 1.94	0.259 ± 0.016
	*lpxL1*^*−*^*rmp*^*−*^	98.86 ± 0.79	0.148 ± 0.011
**NZ98/254**	wild type	119.42 ± 4.71	0.309 ± 0.034
	*lpxL1*^*−*^*rmp*^*−*^	151.99 ± 3.70	0,184 ± 0.011

Using a Zetasizer Nano ZS (Malvern Instruments Ltd., Worcestershire, UK) in triplicate samples (mean ± SD).

*PDI* polydispersity index.

### Immunisation with gonococcal nOMV results in accelerated bacterial clearance in the *N. gonorrhoeae* oestradiol-treated BALB/c mouse infection model compared with 4CMenB and meningococcal nOMV

Three groups of 20 six-week-old female BALB/c mice were immunised subcutaneously between the shoulder blades at 0, 3 and 6 weeks with 250 µL of one of the following: Formulated GC_0817560*lpxL1*^*−*^*rmp*^*−*^ nOMV containing 12.5 µg protein-equivalents of nOMV and 0.75 mg of Al(OH)_3_; or 4CMenB (corresponding to 25 µg of each recombinant protein, 12.5 µg of NZ98/254 dOMV and 0.75 mg Al(OH)_3_); or 250 µL Al(OH)_3_ solution (containing 0.75 µg Al(OH)_3_ in PBS as an adjuvant control). All immunisations were well tolerated. Two weeks after the last immunisation, mice in the dioestrus phase of the oestrous cycle (10 mice per group) were treated with Premarin® (Pfizer) and infected intravaginally with 4.5 × 10^7^ CFU *N. gonorrhoeae* FA1090. Vaginas were swabbed daily to determine the kinetic of the gonococcal infection.

**Fig. 9:**
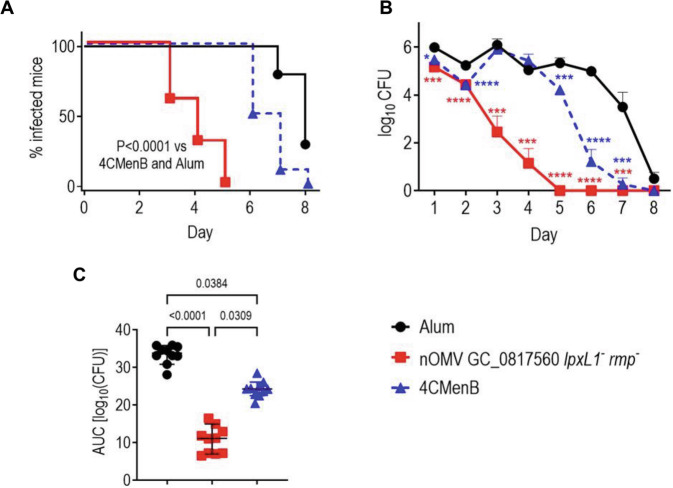
Efficacy of GC_0817560 *lpxL1*^−^*rmp*^−^ nOMV against *N. gonorrhoeae* FA1090 in the mouse vaginal colonisation model. Six week-old female BALB/c mice were immunised with GC_0817560*lpxL1*^−^*rmp*^−^ nOMV, 4CMenB or Al(OH)_3_ (adjuvant control) at 0, 3 and 6 weeks. Two weeks after the last immunisation, 10 mice in each group in the dioestrus phase of the oestrous cycle were treated with Premarin and infected intravaginally with strain FA1090 (4.5 × 10^7^ CFU). **A** Kaplan Meier (KM) analysis showing time to clearance of infection. Groups were compared using the Mantel-Cox log-rank test. The difference between the KM curves was as follows: nOMV GC_0817560*lpxL1*^−^*rmp*^−^ vs. Al(OH)_3_, *P* < 0.0001; 4CMenB vs. Al(OH)_3,_ *P* = 0.0008; nOMV GC_0817560*lpxL1*^−^*rmp*^−^ vs. 4CMenB^,^ *P* < 0.0001. **B** Log_10_ CFU versus time. Comparisons between the two vaccine groups and the adjuvant control group were made by two-way ANOVA and Dunnett’s multiple comparisons test. *, *P* < 0.05; ***, *P* < 0.001; ****, *P* < 0.0001. The colour of the asterisks corresponds to the colour of the lines/symbols of the dataset (red asterisks for nOMV GC_0817560*lpxL1*^−^*rmp*^−^ vs. Al(OH)_3_ and blue asterisks for 4CMenB vs. Al(OH)_3_). **C** Area Under the Curve [log_10_ CFU] analysis. The horizontal lines represent the mean AUC of each group. The three groups were compared using one-way ANOVA (the non-parametric Kruskal–Wallis equality of populations rank test). Pairwise comparisons across groups (*P* values indicated with the graph) were made with Dunn’s multiple comparisons test.

While mice immunised with 4CMenB cleared gonococci faster than mice in the control group immunised with Al(OH)_3_ (*p* = 0.008), mice immunised with gonococcal nOMV cleared gonococcus more rapidly than both 4CMenB-immunised and Al(OH)_3_-immunised mice (*p* < 0.0001; Mantel-Cox log-rank test, *n* = 10 per group) as demonstrated by Kaplan Meier analysis (Fig. 9A). All gonococcal nOMV-immunised mice cleared gonococci by day 5 post challenge, while 100% of 4CMenB-immunised mice were still infected at this time point. It took 8 days for all 4CMenB-immunised mice to clear infection. Reduction in absolute gonococcal bacterial load with time compared with the control group was greater for mice immunised with gonococcal nOMV each day between days 3 and 5 following infection compared with mice immunised with 4CMenB (Fig. 9B). By area under the curve (AUC) analysis of bacterial load with time (Fig. 9C), mice immunised with gonococcal nOMV had a much lower bacterial load than mice immunised with either 4CMenB (*p* = 0.0309) or Al(OH)_3_ (*p* < 0.001), while those immunised with 4CMenB had a lower load than control mice (*p* = 0.0384; Dunn’s multiple comparisons test, *n* = 10 per group).

In order to corroborate and extend these findings, we repeated the gonococcal infection challenge study, including two additional groups: To assess whether an improvement in efficacy could be achieved using gonococcal nOMV derived from the same strain of *N. gonorrhoeae* as the infecting strain, we immunised groups of mice with nOMV produced from FA1090*lpxL1*^*−*^*rmp*^*−*^ as well as GC_0817560*lpxL1*^*−*^*rmp*^*−*^. To explore whether immunisation with nOMV derived from NZ98/254 could clear gonococci more quickly than the NZ98/254 dOMV present in 4CMenB, we included mice immunised with NZ98/254*lpxL1*^*–*^*rmp*^*–*^ nOMV in addition to mice immunised with 4CMenB. Otherwise, the study was conducted as previously, apart from group sizes, which were smaller, with 8–10 mice from each group in the dioestrus phase treated with Premarin before being infected intravaginally with 4 × 10^7^ CFU *N. gonorrhoeae* FA1090.

**Fig. 10:**
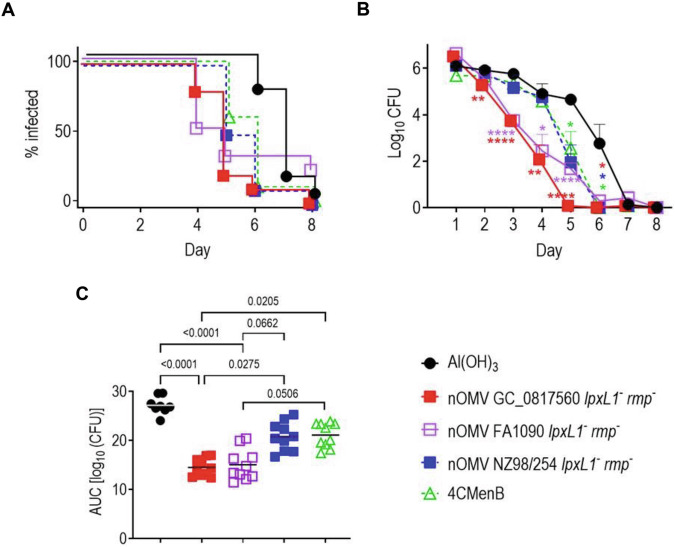
Efficacy of GC_0817560*lpxL1*^*−*^*rmp*^*−*^ nOMV, FA1090*lpxL1*^*−*^*rmp*^*−*^ nOMV, NZ98/254*lpxL1*^*−*^*rmp*^*−*^ nOMV and 4CMenB against *N. gonorrhoeae* FA1090 in the mouse vaginal colonisation model. Six-week-old female BALB/c mice were immunised with three candidate *Neisseria* nOMV vaccines or Al(OH)_3_ (adjuvant control) at 0, 3 and 6 weeks. Two weeks after the last immunisation, 8–10 mice in each group in the dioestrus phase of the oestrous cycle were treated with Premarin and infected intravaginally with strain FA1090 (4 × 10^7^ CFU). **A** Kaplan–Meier analysis showing time to clearance of infection. Groups were compared using the Mantel-Cox log-rank test. GC_0817560*lpxL1*^*–*^*rmp*^*–*^ nOMV, NZ98/254*lpxL1*^*–*^*rmp*^*–*^ nOMV and 4CMenB differed significantly compared to the Al(OH)_3_ group (*P* values of 0.012, 0.023 and 0.028, respectively). The differences between the Al(OH)_3_ group and FA1090*lpxL1*^*–*^*rmp*^*–*^ nOMV group (*P* = 0.64), and 4CMenB group and GC_0817560*lpxL1*^*–*^*rmp*^*–*^ nOMV group (*P* = 0.14) were not significant. **B** Log_10_ CFU versus time. Comparisons between the vaccine groups and the adjuvant control group were made by two-way ANOVA and Dunnett’s multiple comparisons test. *, *P* < 0.05; **, *P* < 0.01; ****, *P* < 0.0001. The colour of the asterisks corresponds to the colour of the lines/symbols of that dataset (i.e. the red, purple, blue and green asterisks correspond to the GC_0817560*lpxL1*^*–*^*rmp*^*–*^, FA1090, NZ98/254 and 4CMenB groups) when compared with the Al(OH)_3_ group. **C** Area Under the Curve [log_10_ CFU] analysis. The horizontal lines represent the mean AUC of each group. The groups were compared using one-way ANOVA (the non-parametric Kruskal–Wallis equality of populations rank test). Pairwise comparisons across groups (*P* values indicated with the graph) were made with Dunn’s multiple comparisons test.

As before, all immunisations were well tolerated. By Kaplan Meier analysis, groups immunised with GC_0817560*lpxL1*^*−*^*rmp*^*−*^ nOMV, NZ98/254*lpxL1*^*−*^*rmp*^*−*^ nOMV and 4CMenB cleared their gonococcal infection significantly more rapidly than mice immunised with Al(OH)_3_ (*p* = 0.012, *p* = 0.023, *p* = 0.028, respectively; Mantel-Cox log-rank test, *n* = 8–10 per group) (Fig. 10A). However, clearance of gonococci from mice immunised with FA1090*lpxL1*^*−*^*rmp*^*−*^ nOMV was not significantly quicker (*p* = 0.64, Mantel-Cox log-rank test, *n* = 8–10 per group). While gonococcal vaginal bacterial load in mice immunised with 4CMenB or NZ98/254*lpxL1*^*–*^*rmp*^*–*^ nOMV did not differ from mice immunised with Al(OH)_3_ until Day 5 post challenge, mice immunised with GC_0817560*lpxL1*^*–*^*rmp*^*–*^ nOMV and FA1090*lpxL1*^*–*^*rmp*^*–*^ nOMV had significantly lower gonococcal bacterial load by Day 2 and Day 3 (Fig. 10B). By AUC analysis, gonococcal bacterial load was again significantly lower in mice immunised with GC_0817560*lpxL1*^*–*^*rmp*^*–*^ nOMV compared with mice immunised with 4CMenB, and to a similar extent lower than mice immunised with NZ98/254*lpxL1*^*–*^*rmp*^*–*^ nOMV (*p* = 0.0205 and *p* = 0.0275 respectively; Dunn’s multiple comparisons test) (Fig. 10C). Mice immunised with either GC_0817560*lpxL1*^*−*^*rmp*^*−*^ nOMV or FA1090*lpxL1*^*−*^*rmp*^*−*^ nOMV had significantly lower AUC values that control mice (*p* < 0.0001 for both), though those immunised with FA1090*lpxL1*^*−*^*rmp*^*−*^ nOMV fell just above the limit of significance when compared with either 4CMenB- (*p* = 0.051) or NZ98/254*lpxL1*^−^*rmp*^−^ nOMV-immunised mice (*p* = 0.066).

### Gonococcal nOMV induces a robust serum anti-gonococcal IgG response compared with 4CMenB

**Fig. 11:**
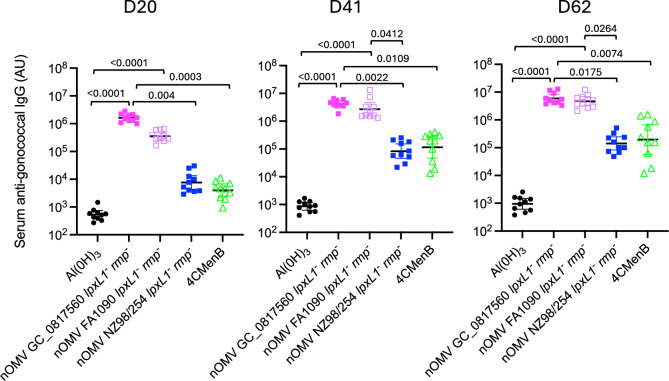
Serum anti-gonococcal IgG of mice immunised with nOMV GC_0817560*lpxL1*^*–*^*rmp*^*–*^, nOMV FA1090*lpxL1*^*–*^*rmp*^*–*^, nOMV NZ98/254*lpxL1*^*–*^*rmp*^*–*^ and 4CMenB. Six-week-old female BALB/c mice were immunised with the four vaccines formulated in Al(OH)_3_ or Al(OH)_3_ alone (adjuvant control, black circles) at 0, 3 and 6 weeks. Mice were bled 3 weeks after each immunisation (Days 20, 41 and 62). Serum anti-gonococcal IgG levels were determined by ELISA for 10 mice per group. ELISA coating substrate was GC_0817560 nOMV. Data were analysed using Kruskal–Wallis and Dunn’s correction for multiple comparison, all significant results are indicated with the adjusted *P* value.

Serum anti-gonococcal IgG was determined for 10 unchallenged mice from each group in the second gonococcal infectious challenge experiment 3 weeks post each of the three doses (days 20, 41 and 62 post first immunisation) by ELISA using plates coated with GC_0817560 nOMV (Fig. 11). At all three time points, anti-gonococcal IgG levels were higher in mice immunised with GC_0817560*lpxL1*^*–*^*rmp*^*–*^ nOMV compared with mice immunised with 4CMenB or NZ98/254*lpxL1*^*–*^*rmp*^*–*^ nOMV (day 20 *P* = 0.0003 and *P* = 0.004, respectively, at day 41, *P* = 0.0109 and *P* = 0.0022 respectively, and at day 62, *P* = 0.0074 and *P* = 0.0175, respectvely). FA1090*lpxL1*^*–*^*rmp*^*–*^ nOMV also induced higher serum IgG antibody titre compared with mice immunised with nOMV NZ98/254*lpxL1*^*–*^*rmp*^*–*^ nOMV after 2 and 3 doses (*P* = 0.0412 and *P* = 0.0264, respectively). Anti-gonococcal IgG significantly increased in all vaccine-immunised groups following two and three immunisations compared with one immunisation (Supplementary Fig. 5), indicating a boosting of antibody levels with a second vaccination.

### Serum anti-gonococcal IgG from mice immunised with *Neisseria* OMV candidate vaccines or 4CMenB is unable to effect complement-mediated killing of *N. gonorrhoeae* FA1090

**Fig. 12:**
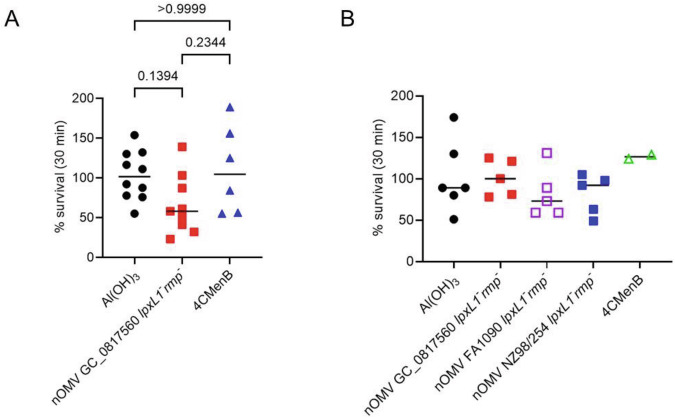
Antibody-dependent complement-mediated killing by sera from mice immunised with *Neisseria* nOMV candidate vaccines or 4CMenB against *N. gonorrhoeae* strain FA1090. Sera were prepared from mice that were not used for challenge experiments following terminal bleeding (**A** represents residual animals following challenge in Fig. 9, and **B** the residual animals following challenge in Fig. 10). Sera were passed through anti-mouse IgM agarose (Sigma) to remove natural IgM that is bactericidal against *N. gonorrhoeae*^31^. Approximately 1000 CFU *N. gonorrhoeae* FA1090 grown to the mid-log phase and suspended in HBSS containing 1 mM each of CaCl_2_ and MgCl_2_ (HBSS^++^) were incubated with IgM-depleted mouse serum (final concentration 33%) and human complement (IgG and IgM-depleted normal human serum (Pel-Freez), final concentration 20%) in a final volume of 70 µL. Aliquots were plated at the start of the assay (*t* = 0 min) and again at 30 min following incubation at 37 °C for min (*t* = 30 min). Plates were incubated at 37 °C for 24 h in an atmosphere enriched with 5% CO_2_. The percentage of CFUs at 30 min relative to 0 min, expressed as a percentage, is shown as survival on the Y-axis. Groups were compared using one-way ANOVA, and pairwise comparisons were made by Dunn’s multiple comparisons test. There were no significant differences in pairwise comparisons across each of the groups in (**B**) (*P* > 0.8).

We performed serum bactericidal assays using sera prepared from terminal bleeds of mice that were not used for challenge. Because sera from adjuvant control mice possessed high levels of bactericidal IgM as reported previously^31^, we depleted IgM by passage of sera through anti-mouse IgM agarose. As shown in Fig. 12, the mean bactericidal activity of the sera from the vaccine groups did not differ from that of the Al(OH)_3_ control group.

## Discussion

The spread of multidrug- and extensively-drug-resistant *N. gonorrhoeae* strains has generated an urgent need for a vaccine against gonorrhoea. A retrospective case-control study among adolescents and young adults in New Zealand vaccinated with *N. meningitidis* dOMV (MeNZB) reported 31% effectiveness against gonorrhoea^14^, supporting the use of an OMV-based approach to develop a vaccine against gonorrhoea. Subsequent observational studies with 4CMenB have found protection against gonorrhoea ranging from 35% to 59%^32,33^ and several prospective randomised clinical trials with 4CMenB are in progress^33^. OMV, as vaccine candidates, are affordable and easy to manufacture. However, for feasible vaccine production yield and to reduce lipid A reactogenicity, detergent-extraction methods have been used to produce dOMV present in current licensed vaccines such as 4CMenB. In the present work, we designed, characterised and studied the efficacy in mice against gonococcal infection of *Neisseria* nOMV produced without the use of detergents from gonococcal strains GC_0817560 and FA1090 and meningococcal strain NZ98/254, all with deleted *lpxL1* and *rmp* genes to reduce reactogenicity and generation of potentially non-protective antibodies.

An ideal gonococcal vaccine needs to be affordable and cross-protective against different strains. A panel of 20 global circulating strains representative of the diversity of five target antigens (LbpA, LbpB, TbpA, TbpB and MtrE) was collated, from which *N. gonorrhoeae* GC_0817560 was selected based on nOMV production. Gonococcal laboratory strain FA1090, used as the challenge strain in the gonococcal mouse infection model, and meningococcal NZ98/254 strain, from which extracted dOMV are a component of the 4CMenB vaccine, were selected as additional gonococcal and meningococcal nOMV production strains.

nOMV are derived from the outer membrane of bacteria, and contain high levels of lipooligosaccharide and other surface-exposed pathogen-associated molecular patterns (PAMPs), which strongly activate innate immunity and may cause unacceptable reactogenicity. Quantification of IL-6 release from monocytes by MAT provides a relevant, regulatory-accepted in vitro method for evaluating the inflammatory and pyrogenic potential of nOMV-based vaccine formulations supporting the safety evaluation of nOMV-derived vaccine candidates^34^. As expected, deletion of *lpxL1* resulted in a pentacylated lipid A, which was confirmed by mass spectrometry and found to induce substantially lower IL-6 release in the MAT than wild-type lipid A, indicating successful attenuation of reactogenicity. Deletion of *rmp* led to the abrogation of Rmp expression and increased nOMV yield.

Immunisation with 4CMenB by the intraperitoneal and subcutaneous routes has previously been demonstrated to accelerate clearance of gonococci in the oestradiol-treated mouse gonococcal vaginal infection model^35^. Consistent with our a priori hypothesis, immunisation with our gonococcal nOMV candidate vaccine from *N. gonorrhoeae* GC_0817560*lpxL1*^*–*^*rmp*^*–*^, via the subcutaneous route, resulted in accelerated clearance of *N. gonorrhoeae* in the mouse infection model compared with immunisation with either Al(OH)_3_ or 4CMenB. This finding of enhanced efficacy in mice corresponded with the induction of higher levels of serum anti-gonococcal IgG compared with 4CMenB. Interestingly, given that gonococcus is a mucosal pathogen, infecting the genital epithelium, efficacy resulted from parenteral immunisation. This finding concurs with the observed effectiveness of 4CMenB against gonorrhoea in humans when delivered parenterally, and suggests that mucosal administration, as used by other investigators^36,37^ may not be necessary for a protective gonococcal vaccine.

Importantly, in our first mouse infection model experiment with GC_0817560*lpxL1*^*–*^*rmp*^*–*^ nOMV, efficacy was elicited against gonococcal infection with a heterologous strain of *N. gonorrhoeae*, FA1090. While GC_0817560 and FA1090 show high core genome conservation, the substantial structural divergence in major outer membrane targets such as PorB (93.4% identity) and TbpB (82.6% identity), combined with the functional absence of LbpA/B in FA1090, confirms that GC_0817560 and FA1090 have highly distinct antigenic repertoires and can be considered heterologous strains. Efficacy was demonstrated both in relation to the percentage of mice infected with time and the total bacterial load.

Since *N. gonorrhoeae* GC_0817560 and FA1090 are phylogenomically distinct among global gonococcal isolates, our findings suggest that our candidate vaccine could have broad reactivity against a range of gonococcal strains. The potential ability of gonococcal nOMV to confer broad protection against different *N. gonorrhoeae* strains should possibly be anticipated given the protection conferred by meningococcal group B OMV-based vaccines in humans across a range of clinical settings. MeNZB and 4CMenB have been shown consistently to have effectiveness in retrospective studies in humans against gonorrhoea across a range of real-world evidence studies^32,33^, presumably caused by genetically diverse strains of *N. gonorrhoeae*. While we acknowledge that we used only a single strain of *N. gonorrhoeae* (FA1090) in the mouse protection studies, in light of the current findings, it is reasonable to speculate that gonococcal nOMV vaccines could afford greater protection against gonorrhoea compared with these meningococcal OMV-based vaccines.

Surprisingly, in the second mouse infection study, there was no improvement in efficacy when nOMV were derived from the homologous FA1090 strain of *N. gonorrhoeae* rather than GC_0817560. Indeed, by Kaplan Meier analysis, clearance of gonococci from mice immunised with FA1090*lpxL1*^*-*^*rmp*^*-*^ nOMV was not significantly quicker than from Al(OH)_3_-immunised mice, although bacterial loads were lower than for these control mice by 3 days after infectious challenge. Based on our available data to date, nOMV derived from GC_0817560 rather than FA1090, appear to constitute a more promising vaccine candidate against gonorrhoea. As nOMV display outer membrane proteins on their outer surface while dOMV have these antigens orientated on their inner surface, better efficacy might be expected with nOMV compared with dOMV. However, nOMV derived from NZ98/254 demonstrated almost identical efficacy to 4CMenB (containing NZ98/254 dOMV) against gonococcal infection in this study. We did not have gonococcal dOMV available to compare efficacy between paired gonococcal nOMV and dOMV.

Gonococcal OMV candidate vaccines based on *N. gonorrhoeae* FA1090 have been investigated by others in the field. Liu and colleagues demonstrated that OMV derived from *N. gonorrhoeae* FA1090 could enhance clearance of FA1090 in the mouse infection model when administered intravaginally^36^ or intranasally^37^ but only when coadministered with interleukin-12-containing microspheres. Recently, Spinsanti and colleagues described the humoral and cellular immune responses induced by an *N. gonorrhoeae* FA1090 OMV candidate vaccine administered by the intraperitoneal route^38^. We are unaware of any publicly available information on the performance of this vaccine in the mouse gonococcal infection model. The candidate was being assessed in a Phase 1/2 clinical trial (Clinical trial.gov NCT05630859, Registration Date 25 November 2022)^39^ but further development was halted when the vaccine failed to meet pre-defined (and, as yet, unspecified) efficacy criteria^40^.

It will be important in future studies to explore the basis of efficacy of GC_0817560*lpxL1*^*–*^*rmp*^*–*^ nOMV both in relation to immune mechanisms and target antigens. In the current study, we have demonstrated robust and boostable anti-gonococcal serum IgG responses following immunisation with both *N. gonorrhoeae* GC_0817560*lpxL1*^*–*^*rmp*^*–*^ nOMV and FA1090*lpxL1*^*–*^*rmp*^*–*^ nOMV. Although efficacy in vivo was noted against *N. gonorrhoeae* FA1090, we did not observe an increase in complement-mediated bactericidal activity of the elicited IgG antibodies. Our data differ from Cuffaro et al.^41^, who demonstrated that mice immunised with gonococcal nOMV elicited bactericidal antibodies that target lipooligosaccharide^41^. While Cuffaro et al. used intact serum, our study used IgM-depleted serum, which may have contributed to the differences in bactericidal activity.

Functionality of these antibodies, breadth of activity against a range of gonococcal strains, as well as the induced T-cell responses, requires further exploration and will, in turn, help understand better what is required for protective immunity against gonorrhoea^16^. *N. gonorrhoeae* GC_0817560 was selected from a panel of representative circulating global gonococcal strains chosen on the basis of the diversity of five potential vaccine antigens, LbpA, LbpB, TbpA, TbpB and MtrE. However, it is currently unclear which of these are targets of protective immunity and what other gonococcal antigens, including gonococcal lipo-oligosaccharide, might also be important for protection. It is both conceivable and plausible that immune responses to a plethora of gonococcal nOMV antigens contribute to efficacy against gonococcal infection in mice. Two recent studies noted that the efficacy of 4CMenB in the mouse vaginal colonisation model does not correlate with serum bactericidal activity^42,43^, suggesting that mechanisms other than antibody-dependent complement-mediated killing may contribute to protection mediated by OMV-based vaccines.

The decision of the United Kingdom^44^ and New York State^33^ in 2025 to recommend 4CMenB for individuals at high risk of gonorrhoea emphasises the public health relevance of the current work. This relevance has only been enhanced by the recent finding from the GoGoVax Study, a randomised, double-blind, placebo-controlled trial of 4CMenB in men who have sex with men^45^, that 4CMenB does not reduce the incidence of *N. gonorrhoeae* infections in this key target population at high risk of gonorrhoea (incidence rate ratio 1.01, 95%CI 0.80, 1.25, *p* = 0.97)^46^. The increased efficacy observed with our gonococcal nOMV candidate vaccine over 4CMenB in mice in this study indicates the potential public health benefit against gonorrhoea and the accompanying burden of antimicrobial resistance from this vaccine. However, efficacy against gonorrhoea needs to be demonstrated in clinical trials in humans. Nevertheless, the prospect of greater protection from a gonococcal nOMV vaccine compared with 4CMenB provides a strong case for the further development of GC_0817560*lpxL1*^*–*^*rmp*^*–*^ nOMV.

In conclusion, we have developed and characterised gonococcal and meningococcal nOMV and demonstrated that the GC_0817560*lpxL1*^*–*^*rmp*^*–*^ nOMV candidate vaccine clears a heterologous gonococcal infection in the mouse vaginal gonorrhoea model more quickly than 4CMenB and a meningococcal nOMV candidate vaccine. Genetically-modified gonococcal nOMV with deleted *lpxL1* and *rmp* are straightforward to produce, well-tolerated and highly immunogenic, inducing a robust serum IgG antibody response in mice when administered parenterally. These findings suggest that GC_0817560*lpxL1*^*–*^*rmp*^*–*^ nOMV could form the basis of a safe and effective vaccine against gonorrhoea. Progression to clinical studies in humans is the clear next step to understanding the potential of gonococcal nOMV as the basis of a protective vaccine.

## Methods

### Gonococcal isolates

A total of 362 global *N. gonorrhoeae* strains from the WHO Collaborating Centre for Gonorrhoea and other Sexually Transmitted Infections in Örebro (Sweden) with available genomic information and containing five key functional antigen-encoding genes (*lbpA, lbpB, tbpA, tbpB* and *mtrE*) were chosen for analysis. Amino acid sequences of these genes were extracted from the genome assemblies using the annotation information. Sequences from each antigen were aligned to each other using MAFFT v7 and a pseudo-maximum likelihood phylogenetic tree built using FastTree v2.1.9^47^. Pairwise distances were calculated for all pairs of genomes in the five protein-based trees. The overall distance between each pair of genomes was defined as the sum of distances for each antigen tree, considering equal weights for each of the trees. Multidimensional scaling (MDS) was used to project these distances. K-means clustering was used to define clusters based on the MDS variables and define centroids (points in the projection space minimising the sum of Euclidean distances to all members of the cluster). The strain closest to each of the centroids was selected as representative of the cluster. In total, 20 representative strains, which together span the maximum amount of projected variation among all the strains, were selected. Typing information was obtained from the assemblies using Pathogenwatch^30^.

Available short-read Illumina sequencing data for the 362 strains were mapped against the *N. gonorrhoeae* FA1090 reference genome (NCBI accession NC_002946.2; 2,153,922 bp) using SMALT v0.7.4 (http://www.sanger.ac.uk/science/tools/smalt-0), and variants were called as previously described^48^. Single nucleotide polymorphisms (SNPs) were called using SNP-sites^49^. The resulting alignment of 57,310 polymorphic sites was used for phylogenetic reconstruction using FastTree v2.1.9 with the GTR substitution model. The 20 strains representative of the genomic diversity of the starting collection were mapped onto the genome-wide and antigen trees using iToL v3^50^ and Microreact^51^.

A minimum spanning tree was also generated using the GrapeTree plugin on PubMLST^52,53^. This tool compares allelic profiles, clustering gonococci sharing alleles across the core genome. GrapeTree was run by selecting the *N. gonorrhoeae* cgMLST v1.0 scheme and including a globally representative *N. gonorrhoeae* collection consisting of 8738 isolates dating from 1928 to 2019.

The sequences of the *lbpA, lbpB, tbpA, tbpB, mtrE* and *porB* genes were identified and extracted from the genome assembly of *N. gonorrhoeae* FA1090 (NCBI accession NC_002946.2), the selected *N. gonorrhoeae* GC_0817560 strain (ENA run accession ERR349896, assembly available from PubMLST under id 31560), and from the *N. meningitidis* NZ98/254 strain from 4CMenB (ENA run accession ERR1134899, assembly available from PubMLST under id 34542). The coverage and identity of the nucleotide and protein sequences of each gene were compared among the three strains using Web BLAST (blastn and blastp)^54^. The *N. gonorrhoeae* GC_0817560 and FA1090 strains were also compared genome-wise based on 1542 core genes using Pathogenwatch^30^.

### Gonococcal growth conditions for screening

Gonococcal strains were grown at 37 °C with humidified 5% CO_2_-enriched atmosphere on GC agar base (BD Difco, France) supplemented with Vitox (Oxoid, UK) and in GC broth (15 g Protease peptone N˚ 3, 4 g K_2_HPO_4_, 1 g KH_2_PO_4_, 5 g NaCl per litre, pH 7.2) containing 10 ml of Kellogg’s supplement I (40% glucose, 1% glutamine and 0.2% of thiamine pyrophosphate).

### Genetic manipulation

Synthetic genes were designed containing between 500 bp and 600 bp homologous to the upstream and downstream sequences of the *N. gonorrhoeae lpxL1* locus (lpxL1_KO) and *rmp* locus (rmp_KO) with a kanamycin or chloramphenicol resistance cassette in place of *lpxL1* and *rmp*, respectively (GeneArt, Life Technologies Limited, UK). Primers used to amplify or modify synthetic genes are in Table 5. Genes were amplified using primers lpxL1.F and lpxL1.R or Rmp.F and Rmp.R, respectively, and used to transform GC_0817560 and FA1090 as previously described^55^. The *rmp* deletion was introduced after the *lpxL1* deletion, resulting in two genetically modified strains (*lpxL1*^*–*^ and *lpxL1*^*–*^*rmp*^*–*^) confirmed by sequencing (Source BioScience, Nottingham, UK).

**Table 5:** Primer sequences used to generate genetically modified *Neisseria* strains

Primer Name	Squence
lpxL1.F	CTGATTCAACACGGTTTGCT
lpxL1.R	TCTGTAATTCTGTGTAGCGG
Rmp.F	CTGAATGCCGATTTCTTCTTCC
Rmp.R	ACCATATCCGGCATACAGTG
lpxL1long1.R	CAGCGATTATCAAATGAAATCTGTTCATATAATCTGCCATTTCGCATTTAAAAAAACAAACAGGAGTTTCGACTCGAAACGCCTGATTTGTTTTGTAA
RmpShort.R	CCGTACTATTGTACTGTCTGCG
ModlpxL1.R	CAGCGATTATCAAATGAAATCTG

Downstream regions of *N. meningitidis lpxL1* and *rmp* loci exhibit significant divergence from corresponding gonococcal sequences. Synthetic genes were subsequently engineered to enhance homology with the downstream meningococcal loci. For the modification of lpxL1_KO, a synthetic gene was amplified using lpxL1.F and lpxL1long1.R, obtaining a new gene with 80 bp homology to the downstream region of the *N. meningitidis* NZ98/254 *lpxL1* locus. To generate the modified *N. meningitidis* rmp_KO gene, a synthetic gene was amplified using Rmp.F and RmpShort.R, obtaining a gene with 280 bp homologous to the downstream meningococcal *rmp* locus. Genes were amplified using primer lpxL1.F and ModlpxL1.R or Rmp.F and RmpShort.R, respectively and used to transform NZ98/254. For the generation of bacterial strains with two gene deletions, *lpxL1* deletion was introduced after the *rmp* deletion. All gene deletions were confirmed by whole-genome sequencing (Source BioScience, Nottingham, UK).

### Production of nOMV

Overnight cultures of gonococcal or meningococcal strains plated on GC agar were used to inoculate GC media to an OD_600_ of 0.10 and cultured at 37 °C and 180 rpm with 5% CO_2_ or 0.042% sodium bicarbonate until mid-logarithmic phase. Subsequently, new flasks were inoculated at an OD_600_ of 0.05 and incubated overnight to reach the stationary phase at an OD_600_ of approximately 1.0. Kanamycin and chloramphenicol were used in growth media for genetically manipulated strains at final concentrations of 50 μg/mL and 1.5 to 5 μg/mL, respectively. Culture supernatants were collected following 10 min centrifugation at 6000 × *g* to pellet cells, followed by 0.22 µm filtration. nOMV were concentrated by ultracentrifugation at 163,800 × *g* for 3 h at 4 °C (Optimal L-100 XP, Beckman Coulter), resuspended in phosphate buffered saline (PBS) followed by 0.22 µm filtration and stored at 4 °C or −80 °C. nOMV were quantified for total protein content using the Lowry assay (DC protein assay, Bio-Rad, USA) according to the manufacturer’s instructions. Measurements were performed at two different dilutions, each in triplicate.

### SDS-PAGE and mass spectrometry

Eight µg protein-equivalents of nOMV were denatured for 5 min at 95 °C in Laemmli sample buffer (Bio-Rad, UK) with 5% DTT (Sigma-Aldrich). Proteins were separated on 4–12% polyacrylamide gradient gels (NuPAGE Bis-Tris gels, Invitrogen, USA) using MES buffer and gels were stained with Coomassie Blue. Protein bands were excised from the gel, in-gel digested with trypsin and identified by LC-MS/MS using a Bruker Esquire HTC ESI-ion trap mass spectrometer fitted with a standard ion source in positive ion mode as described previously^56^.

### Lipid A MALDI-TOF/TOF analysis

Lipid A was precipitated from bacteria resuspended in PBS at an OD_600_ of 1 by mild acid hydrolysis with 1% acetic acid for 6 h at 100 °C, centrifuged at 14,000 × *g* for 15 min, washed twice with water and dried with an SpeedVac vacuum concentrator (Thermo Fisher Scientific). Before analysis, samples were resuspended in 50 μL chloroform/methanol at a 4:1 ratio and mixed with an equal volume of Super DHB solution (Sigma-Aldrich, USA). Two microliters of each sample were spotted directly onto the plates, and data were acquired in positive reflectron mode (*m/z* 800–2000) using a 4800plus MALDI-TOF/TOF (AB Sciex) mass spectrometer and using 4000 Series Explorer Software v.3.5.3 (Applied Biosystems). External calibration was performed using Pepmix1 (Laser BioLabs).

### Transmission electron microscopy

Ten microliters of 0.1 mg/mL nOMV suspensions were applied to glow-discharged carbon-coated 300 mesh copper grids, quickly blotted, stained with 2% uranyl acetate for 10 s, blotted and air dried. Grids were imaged in an FEI Tecnai12 transmission electron microscope operated at 120 kV using a Gatan OneView camera.

### Dynamic light scattering

nOMV were diluted 1:100 in water, and the mean diameter and polydispersion index (PDI) were analysed by dynamic light scattering (DLS) using a Zetasizer Nano ZS (Malvern Instruments Ltd., UK) in triplicate samples. The angle for detection of scattered light was set to 173°, and measurements were performed at 25 °C.

### Monocyte activation test (MAT)

PBMCs from three donors were separated from whole blood using Leucosep tubes containing 15 mL of Lymphoprep (Axis Shield, UK) by centrifugation at 1000 × *g* for 13 min. Cells were incubated for 5 min with RBC lysis solution (Qiagen, Germany), centrifuged and resuspended in RPMI-1640 Medium containing 1% penicillin/streptomycin, 2 mM L-glutamine and 10% heat-inactivated foetal bovine serum (Sigma-Aldrich, USA). Cells, 2 × 10^5^ cells/well, were stimulated with 10-fold serial dilutions of nOMV (0.0001–1000 ng/ml final concentration) or PBS in duplicate wells for 4 h at 37 °C. Supernatants were recovered by centrifugation at 400 × *g* and stored at –80 °C until analysis.

IL-6 release into supernatants following stimulation of PBMCs was measured by ELISA. Plates were coated overnight at 4 °C with anti-human IL-6 antibody (eBioscience, USA) and incubated with supernatants from PBMC stimulations, diluted 1:4 with PBS, for 2 h at room temperature. Plates were washed six times with PBS containing 0.05% Tween 20 after each incubation. Recombinant human IL-6 (eBioscience, USA) was used as the standard. IL-6 was detected using biotin-labelled anti-human IL-6 antibody (eBioscience, USA) for 1 h at room temperature, followed by 30 min incubation with avidin-HPR. After the addition of TMB substrate, plates were incubated for 7 min at room temperature, the reaction was stopped with 2 M sulphuric acid and plates were immediately read at 450 nm.

### Formulation of nOMV

Prior to use in animal studies, stock nOMV preparations, typically at 1–4 mg/mL nOMV in PBS, were formulated by mixing overnight at 4 °C with aluminium hydroxide (Al(OH)_3_) (Alhydrogel, Croda) and PBS to a final concentration of 50 µg/mL nOMV and 3 mg/mL Al(OH)_3_.

### Mouse vaginal colonisation model

Use of animals in this study was performed in strict accordance with the recommendations in the Guide for the Care and Use of Laboratory Animals of the National Institutes of Health. The protocol (PROTO202000074) was approved by the Institutional Animal Care and Use Committee (IACUC) at the University of Massachusetts Medical School. The BALB/c mouse model of vaginal colonisation described by Jerse was used^57^.

Two weeks after the last immunisation, mice in the dioestrus phase of the oestrous cycle were started on treatment (that day) with 0.1 mg Premarin (Pfizer; a mixture of sodium oestrone sulphate and sodium equilin sulphate also containing sodium sulphate conjugates of 17α-dihydroequilin, 17α-oestradiol and 17β-dihydroequilin) in 200 µL of water, given subcutaneously on each of three days; −2, 0 and +2 days (before, the day of and after gonococcal inoculation) to prolong the oestrus phase of the reproductive cycle and promote susceptibility to *N. gonorrhoeae* infection. Antibiotics (vancomycin and streptomycin), ineffective against *N. gonorrhoeae*, were also used to reduce competitive microflora^58^. Mice were infected on Day 0 with *N. gonorrhoeae* FA1090 (inoculum specified for each experiment). Vaginas were swabbed daily, with swabs plated on chocolate agar supplemented with antibiotics (vancomycin, colistin, nystatin, trimethoprim and streptomycin), which suppresses vaginal flora but permits growth of gonococci, to enumerate gonococcal colony forming units (CFU).

Control and vaccine groups were kept in separate cages. Different operators performed the immunisations and infections, the vaginal swabbing and the colony counting. As such, mice were assigned a number and plates were labelled by mouse number and counted using an automated colony counter. The person performing the vaginal swabbing was blinded. No anaesthesia was used while administering subcutaneous injections, colonising mouse vaginas, collecting swabs or obtaining cheek bleeds. For terminal cardiac bleeds, mice were administered isoflurane anaesthesia. Mice were euthanised by carbon dioxide narcosis followed by cervical dislocation.

### ELISA

Levels of serum IgG were assessed using an in-house standardised ELISA. Ten mice per group were assayed, 3-weeks after the first, second and third immunisation with *Neisseria* nOMV, 4CMenB or Al(OH)_3_. Nunc MaxiSorp ELISA plates (Thermo Fisher) were coated with 2 µg/mL of wild-type GC_0817560 nOMV in Dulbecco’s PBS and incubated overnight at 4 °C. Plates were then washed 6 times with 0.05% PBS-Tween and blocked with 200 µL/well of 1% BSA in PBS at 37 °C for 2 h. After washing 6 times with 0.05% PBS-Tween, murine serum samples, standard and QC controls were diluted in 1% BSA in PBS and transferred to the plate in duplicate with 100 µL/well for an overnight incubation at 4 °C. Plates were then washed 6 times with 0.05% PBS-Tween, and 100 µL/well of secondary antibody (Southern Biotech) was added (1:4,000) in 1% BSA in PBS for 2 h at room temperature. After washing 6 times with 0.05% PBS-Tween, plates were developed with 100 µL/well of 3,3’,5,5’-Tetramethylbenzidine (TMB, MP BIOMEDICALS) at room temperature until the top standard reached an optical density (OD) at 630 nm of 0.9. The reaction was stopped by adding 50 µL/well of H_2_SO_4_ 1 M. The OD at 450 nm and 630 nm was measured using an ELx808 absorbance reader (BioTek) via Gen5 ELISA software v3.09 (BioTek).

### Serum bactericidal assay

Antibody-dependent complement-mediated killing was assessed by serum bactericidal assay (SBA). Sera were prepared from mice that were not used for challenge experiments following terminal bleeding. Sera were passed through anti-mouse IgM agarose (Sigma) to remove natural IgM that is bactericidal against *N. gonorrhoeae*^31^. Approximately 1000 CFU *N. gonorrhoeae* FA1090 grown to the mid-log phase and suspended in HBSS containing 1 mM each of CaCl_2_ and MgCl_2_ (HBSS^++^) were incubated with IgM-depleted mouse serum (final concentration 33%) and human complement (IgG- and IgM-depleted normal human serum (Pel-Freez), final concentration 20%) in a final volume of 70 µL. Aliquots were plated at the start of the assay (*t* = 0 min) and again at 30 min following incubation at 37 °C (*t* = 30 min). Plates were incubated at 37 °C for 24 h in an atmosphere enriched with 5% CO_2_. The percentage of CFU at 30 min relative to 0 min was expressed as the percentage survival.

### Statistical analysis

Experiments that compared clearance of *N. gonorrhoeae* in independent groups of mice estimated and tested three characteristics of the data^59^: time to clearance, longitudinal trends in mean log_10_ CFU and the cumulative CFU as area under the curve (AUC). Median time to clearance was estimated using Kaplan-Meier survival curves. Times to clearance were compared between groups using the Mantel-Cox log-rank test. Mean log_10_ CFU trends over time were compared between groups using two-way ANOVA and Dunnett’s multiple comparisons test. The mean AUC (log_10_ CFU versus time) was computed for each mouse to estimate the bacterial burden over time (cumulative infection). The means under the curves were compared between groups using one-way ANOVA with Dunn’s multiple comparisons test. Power calculations estimated that 8 mice per group would detect a 25% decrease in AUC in the vaccine groups compared to the control group (assuming a standard deviation of 5) with an alpha (type I error, or false positive rate) of 0.05 and Power (1-β, where β is the type II error, or false negative rate) of 80%.

Serum IgG ELISA data were analysed in GraphPad Prism 10 (version 10.6.1) using the Kruskal–Wallis test with multiple comparisons, comparing each column with every other column, and Dunn’s multiple comparison for correction. All significant (*P* < 0.05) results are indicated in the graph with the adjusted *P* value. Data from SBA were compared using one-way ANOVA, and pairwise comparisons were made by Dunn’s multiple comparisons test.

## Supplementary information

Supplement Information

## Data Availability

All data supporting the findings of this study are contained within the article and its supplementary content. The raw data can be obtained from the corresponding author upon request.

## References

[1] World Health Organization. *Global Progress Report on HIV, Viral Hepatitis and Sexually Transmitted Infections, 2021*. https://www.who.int/publications/i/item/9789240027077 (2021).

[2] Jensen, J. S. & Unemo, M. Antimicrobial treatment and resistance in sexually transmitted bacterial infections. *Nat. Rev. Microbiol.* **22**, 435–450 (2024). 38509173 10.1038/s41579-024-01023-3

[3] Cohen, M. S. et al. Reduction of concentration of HIV-1 in semen after treatment of urethritis: implications for prevention of sexual transmission of HIV-1. *AIDSCAP Malawi Res. Group. Lancet* **349**, 1868–1873 (1997). 10.1016/s0140-6736(97)02190-9 9217758

[4] Unemo, M. & Jensen, J. S. Antimicrobial-resistant sexually transmitted infections: gonorrhoea and *Mycoplasma genitalium*. *Nat. Rev. Urol.* **14**, 139–152 (2017). 28072403 10.1038/nrurol.2016.268

[5] Maatouk, I. et al. Antimicrobial resistance in *Neisseria gonorrhoeae* in nine sentinel countries within the World Health Organization Enhanced Gonococcal Antimicrobial Surveillance Programme (EGASP), 2023: a retrospective observational study. *Lancet Reg. Health West Pac.* **61**, 101663 (2025). 40922809 10.1016/j.lanwpc.2025.101663 PMC12414356

[6] Unemo, M. et al. WHO global gonococcal antimicrobial surveillance programmes, 2019-22: a retrospective observational study. *Lancet Microbe* **6**, 101181 (2025). 41015046 10.1016/j.lanmic.2025.101181 PMC12715682

[7] World Health Organization. *WHO Bacterial Priority Pathogens List, 2024: Bacterial Pathogens of Public Health Importance to Guide Research, Development and Strategies to Prevent and Control Antimicrobial Resistance*. https://www.who.int/publications/i/item/9789240093461 (2024).

[8] Ross, J. D. C. et al. Oral gepotidacin for the treatment of uncomplicated urogenital gonorrhoea (EAGLE-1): a phase 3 randomised, open-label, non-inferiority, multicentre study. *Lancet* **405**, 1608–1620 (2025). 40245902 10.1016/S0140-6736(25)00628-2

[9] Luckey, A. et al. Zoliflodacin versus ceftriaxone plus azithromycin for treatment of uncomplicated urogenital gonorrhoea: an international, randomised, controlled, open-label, phase 3, non-inferiority clinical trial. *Lancet* **407**, 147–160 (2026). 41391465 10.1016/S0140-6736(25)01953-1 PMC12784215

[10] Greenberg, L. et al. Gonococcal vaccine studies in Inuvik. *Can. J. Public Health* **65**, 29–33 (1974). 4205640

[11] Boslego, J. W. et al. Efficacy trial of a parenteral gonococcal pilus vaccine in men. *Vaccine* **9**, 154–162 (1991). 1675029 10.1016/0264-410x(91)90147-x

[12] Rice, P. A., Shafer, W. M., Ram, S. & Jerse, A. E. *Neisseria gonorrhoeae*: drug resistance, mouse models, and vaccine development. *Annu. Rev. Microbiol.* **71**, 665–686 (2017). 10.1146/annurev-micro-090816-093530 28886683

[13] Edwards, J. L., Jennings, M. P., Apicella, M. A. & Seib, K. L. Is gonococcal disease preventable? The importance of understanding immunity and pathogenesis in vaccine development. *Crit. Rev. Microbiol.* **42**, 928–941 (2016). 26805040 10.3109/1040841X.2015.1105782 PMC4958600

[14] Petousis-Harris, H. et al. Effectiveness of a group B outer membrane vesicle meningococcal vaccine against gonorrhoea in New Zealand: a retrospective case-control study. *Lancet* **390**, 1603–1610 (2017). 28705462 10.1016/S0140-6736(17)31449-6

[15] Jerse, A. E., Bash, M. C. & Russell, M. W. Vaccines against gonorrhea: current status and future challenges. *Vaccine* **32**, 1579–1587 (2014). 24016806 10.1016/j.vaccine.2013.08.067 PMC4682887

[16] Belcher, T., Rollier, C. S., Dold, C., Ross, J. D. C. & MacLennan, C. A. Immune responses to *Neisseria gonorrhoeae* and implications for vaccine development. *Front. Immunol.* **14**, 1248613 (2023). 10.3389/fimmu.2023.1248613 PMC10470030 37662926

[17] Hedges, S. R., Mayo, M. S., Mestecky, J., Hook, E. W. 3rd & Russell, M. W. Limited local and systemic antibody responses to *Neisseria gonorrhoeae* during uncomplicated genital infections. *Infect. Immun.* **67**, 3937–3946 (1999). 10417159 10.1128/iai.67.8.3937-3946.1999 PMC96675

[18] van der Pol, L., Stork, M. & van der Ley, P. Outer membrane vesicles as platform vaccine technology. *Biotechnol. J.* **10**, 1689–1706 (2015). 26912077 10.1002/biot.201400395 PMC4768646

[19] Micoli, F. & MacLennan, C. A. Outer membrane vesicle vaccines. *Semin. Immunol.* **50**, 101433 (2020). 33309166 10.1016/j.smim.2020.101433

[20] Obiero, C. W. et al. A phase 2a randomized study to evaluate the safety and immunogenicity of the 1790GAHB generalized modules for membrane antigen vaccine against *Shigella sonnei* administered intramuscularly to adults from a shigellosis-endemic country. *Front. Immunol.* **8**, 1884 (2017). 10.3389/fimmu.2017.01884 PMC5763125 29375556

[21] Koeberling, O., Giuntini, S., Seubert, A. & Granoff, D. M. Meningococcal outer membrane vesicle vaccines derived from mutant strains engineered to express factor H binding proteins from antigenic variant groups 1 and 2. *Clin. Vaccin. Immunol.* **16**, 156–162 (2009). 10.1128/CVI.00403-08 PMC2643535 19109451

[22] van de Waterbeemd, B. et al. Improved OMV vaccine against *Neisseria meningitidis* using genetically engineered strains and a detergent-free purification process. *Vaccine* **28**, 4810–4816 (2010). 20483197 10.1016/j.vaccine.2010.04.082

[23] Kulshin, V. A. et al. Structural characterization of the lipid A component of pathogenic *Neisseria meningitidis*. *J. Bacteriol.* **174**, 1793–1800 (1992). 1548229 10.1128/jb.174.6.1793-1800.1992 PMC205780

[24] van der Ley, P. et al. Modification of lipid A biosynthesis in *Neisseria meningitidis* lpxL mutants: influence on lipopolysaccharide structure, toxicity, and adjuvant activity. *Infect. Immun.* **69**, 5981–5990 (2001). 11553534 10.1128/IAI.69.10.5981-5990.2001 PMC98725

[25] Rice, P. A., Vayo, H. E., Tam, M. R. & Blake, M. S. Immunoglobulin G antibodies directed against protein III block killing of serum-resistant *Neisseria gonorrhoeae* by immune serum. *J. Exp. Med.* **164**, 1735–1748 (1986). 3095479 10.1084/jem.164.5.1735 PMC2188443

[26] Gulati, S. et al. Antibody to reduction modifiable protein increases the bacterial burden and the duration of gonococcal infection in a mouse model. *J. Infect. Dis.* **212**, 311–315 (2015). 25596304 10.1093/infdis/jiv024 PMC4565997

[27] Leuzzi, R. et al. *Neisseria gonorrhoeae* PIII has a role on NG1873 outer membrane localization and is involved in bacterial adhesion to human cervical and urethral epithelial cells. *BMC Microbiol.* **13**, 251 (2013). 24206788 10.1186/1471-2180-13-251 PMC4226279

[28] Freixeiro, P. et al. High resolution clear native electrophoresis (hrCNE) allows a detailed analysis of the heterotrimeric structure of recombinant *Neisseria meningitidis* porins inserted into liposomes. *J. Proteome Res.* **12**, 777–784 (2013). 23259616 10.1021/pr3008573

[29] Grizot, S. & Buchanan, S. K. Structure of the OmpA-like domain of RmpM from *Neisseria meningitidis*. *Mol. Microbiol.* **51**, 1027–1037 (2004). 14763978 10.1111/j.1365-2958.2003.03903.x

[30] Sanchez-Buso, L. et al. A community-driven resource for genomic epidemiology and antimicrobial resistance prediction of *Neisseria gonorrhoeae* at Pathogenwatch. *Genome Med.* **13**, 61 (2021). 33875000 10.1186/s13073-021-00858-2 PMC8054416

[31] Gulati, S. et al. Preclinical efficacy of a lipooligosaccharide peptide mimic candidate gonococcal vaccine. *mBio* **10**, 10.1128/mBio.02552-19 (2019). 10.1128/mBio.02552-19 PMC6831779 31690678

[32] Wang, B., Mohammed, H., Andraweera, P., McMillan, M. & Marshall, H. Vaccine effectiveness and impact of meningococcal vaccines against gonococcal infections: a systematic review and meta-analysis. *J. Infect.* **89**, 106225 (2024). 38986746 10.1016/j.jinf.2024.106225

[33] De Wals, P., Bui, Y. G. & Desjardins, M. Effectiveness of the four-component protein-based meningococcal vaccine against *Neisseria gonorrhoeae* infections: mounting evidence and public health implications for Canada. *Can. Commun. Dis. Rep.* **51**, 312–318 (2025). 40919220 10.14745/ccdr.v51i08a04 PMC12410833

[34] Schindler, S., von Aulock, S., Daneshian, M. & Hartung, T. Development, validation and applications of the monocyte activation test for pyrogens based on human whole blood. *ALTEX* **26**, 265–277 (2009). 20383472 10.14573/altex.2009.4.265

[35] Leduc, I. et al. The serogroup B meningococcal outer membrane vesicle-based vaccine 4CMenB induces cross-species protection against *Neisseria gonorrhoeae*. *PLoS Pathog.* **16**, e1008602 (2020). 33290434 10.1371/journal.ppat.1008602 PMC7748408

[36] Liu, Y. et al. Experimental vaccine induces Th1-driven immune responses and resistance to *Neisseria gonorrhoeae* infection in a murine model. *Mucosal Immunol.* **10**, 1594–1608 (2017). 28272393 10.1038/mi.2017.11 PMC5591041

[37] Liu, Y. et al. Microencapsulated IL-12 drives genital tract immune responses to intranasal gonococcal outer membrane vesicle vaccine and induces resistance to vaginal infection with diverse strains of *Neisseria gonorrhoeae*. *mSphere* **8**, e0038822 (2023). 36537786 10.1128/msphere.00388-22 PMC9942569

[38] Spinsanti, M. et al. A novel GMMA-based gonococcal vaccine demonstrates functional immune responses in mice. *NPJ Vaccines* **10**, 146 (2025). 40617825 10.1038/s41541-025-01190-1 PMC12228689

[39] ClinicalTrials.gov. *Safety and efficacy of GSK Neisseria gonorrhoeae GMMA (NgG) investigational vaccine when administered to healthy adults 18 to 50 years of age* (2022). https://clinicaltrials.gov/search?term=NCT05630859 (2026).

[40] Fierce Biotech. *GSK axes vaccine from $2.1B deal, switching to preclinical successor over ‘increased competition’*, https://www.fiercebiotech.com/biotech/gsk-axes-vaccine-21b-deal-switching-preclinical-successor-over-increased-competition (2024).

[41] Cuffaro, R. et al. Contribution of the different *Neisseria gonorrhoeae* lipooligosaccharide structural variants to functional responses elicited by GMMA outer membrane vesicles. *NPJ Vaccines* **10**, 223 (2025). 10.1038/s41541-025-01271-1 PMC12575615 41168226

[42] Zhu, W. et al. Protection against N. gonorrhoeae induced by OMV-based meningococcal vaccines are associated with cross-species directed humoral and cellular immune responses. *Front. Immunol.* **16**, 1539795 (2025). 40292302 10.3389/fimmu.2025.1539795 PMC12021806

[43] Zeppa, J. J. et al. Meningococcal vaccine 4CMenB elicits a robust cellular immune response that targets but is not consistently protective against *Neisseria gonorrhoeae* during murine vaginal infection. *mSphere* **10**, e0094024 (2025). 40237483 10.1128/msphere.00940-24 PMC12108064

[44] Ladhani, S. N., Ramsay, M. E. & Fifer, H. What can be learnt from the world’s first national vaccination programme against gonorrhoea. *Lancet Infect. Dis.* **25**, 1168–1170 (2025). 40848731 10.1016/S1473-3099(25)00468-2

[45] Seib, K. L. et al. Multicentre double-blind randomised placebo-controlled trial evaluating the efficacy of the meningococcal B vaccine, 4CMenB (Bexsero), against *Neisseria gonorrhoeae* infection in men who have sex with men: the GoGoVax study protocol. *BMJ Open* **14**, e081675 (2024). 38626958 10.1136/bmjopen-2023-081675 PMC11029339

[46] Seib, K. L. et al. *Meningococcal B (4CMenB) vaccination for the prevention of gonorrhea in men who have sex with men.**CROI 2026 Conference on Retroviruses and Opportunistic Infections.* https://www.croiconference.org/wp-content/uploads/sites/2/resources/2026/croi2026-abstract-ebook.pdf (2026).

[47] Price, M. N., Dehal, P. S. & Arkin, A. P. FastTree 2-approximately maximum-likelihood trees for large alignments. *PLoS ONE* **5**, e9490 (2010). 20224823 10.1371/journal.pone.0009490 PMC2835736

[48] Sanchez-Buso, L. et al. The impact of antimicrobials on gonococcal evolution. *Nat. Microbiol.* **4**, 1941–1950 (2019). 31358980 10.1038/s41564-019-0501-y PMC6817357

[49] Page, A. J. et al. SNP-sites: rapid efficient extraction of SNPs from multi-FASTA alignments. *Micro. Genom.* **2**, e000056 (2016). 10.1099/mgen.0.000056 PMC5320690 28348851

[50] Letunic, I. & Bork, P. Interactive tree of life (iTOL) v3: an online tool for the display and annotation of phylogenetic and other trees. *Nucleic Acids Res.* **44**, W242–W245 (2016). 27095192 10.1093/nar/gkw290 PMC4987883

[51] Argimon, S. et al. Microreact: visualizing and sharing data for genomic epidemiology and phylogeography. *Micro. Genom.* **2**, e000093 (2016). 10.1099/mgen.0.000093 PMC5320705 28348833

[52] Jolley, K. A., Bray, J. E. & Maiden, M. C. J. Open-access bacterial population genomics: BIGSdb software, the PubMLST.org website and their applications. *Wellcome Open Res.* **3**, 124 (2018). 30345391 10.12688/wellcomeopenres.14826.1 PMC6192448

[53] Zhou, Z. et al. GrapeTree: visualization of core genomic relationships among 100,000 bacterial pathogens. *Genome Res.* **28**, 1395–1404 (2018). 30049790 10.1101/gr.232397.117 PMC6120633

[54] Johnson, M. et al. NCBI BLAST: a better web interface. *Nucleic Acids Res.* **36**, W5–W9 (2008). 18440982 10.1093/nar/gkn201 PMC2447716

[55] Dillard, J. P. Genetic manipulation of *Neisseria gonorrhoeae*. in *Current Protocols in Microbiology*, Ch 4, 10.1002/9780471729259.mc04a02s23 (2011). 10.1002/9780471729259.mc04a02s23 PMC4549065 22045584

[56] Chalk, R. et al. High-throughput mass spectrometry applied to structural genomics. *Chromatography* **1**, 159–175 (2014).

[57] Jerse, A. E. Experimental gonococcal genital tract infection and opacity protein expression in estradiol-treated mice. *Infect. Immun.* **67**, 5699–5708 (1999). 10531218 10.1128/iai.67.11.5699-5708.1999 PMC96944

[58] Jerse, A. E. et al. Estradiol-treated female mice as surrogate hosts for *Neisseria gonorrhoeae* genital tract infections. *Front. Microbiol.* **2**, 107 (2011). 21747807 10.3389/fmicb.2011.00107 PMC3129519

[59] Gulati, S. et al. Immunization against a saccharide epitope accelerates clearance of experimental gonococcal infection. *PLoS Pathog.* **9**, e1003559 (2013). 24009500 10.1371/journal.ppat.1003559 PMC3757034

[60] Harrison, O. B. et al. *Neisseria gonorrhoeae* population genomics: use of the gonococcal core genome to improve surveillance of antimicrobial resistance. *J. Infect. Dis.* **222**, 1816–1825 (2020). 32163580 10.1093/infdis/jiaa002 PMC7653085

