# Supplementary material for: Pre-clinical efficacy of a candidate outer membrane vesicle gonococcal vaccine in comparison with 4CMenB
Source: NPJ Vaccines. 2026 Jun 8;11:126. doi: 10.1038/s41541-026-01491-z (PMC13315605; doi:10.1038/s41541-026-01491-z)
Supplement: Supplementary file 1 — Supplement Information [file 41541_2026_1491_MOESM1_ESM.pdf]

Supplementary Table 1 Identity between vaccine strain GC_0817560 and mouse challenge strain FA1090. In FA1090, *lbpA* is disrupted and *lbpB* is absent.

	**Nucleotide level**		**Protein level**	
**Protein**	**Coverage (%)**	**Identity**	**Coverage (%)**	**Identity**
**MtrE**	100	1397/1404(99.5%)	100	464/467(99.4%)
**TbpA**	100	2684/2745(97.8%)	100	881/914(96.4%)
**TbpB**	100	1921/2126(90.4%)	100	584/707(82.6%)
**PorB**	100	1008/1050(96.0%)	100	326/349(93.4%)

Supplementary Table 2 Identity between vaccine strain GC_0817560 and the 4CMenB NZ98/254 strain.

	Nucleotide level		Protein level	
**Protein**	**Coverage (%)**	**Identity**	**Coverage (%)**	**Identity**
**MtrE**	100	1327/1404(94.5%)	100	451/467(96.6%)
**TbpA**	100	2616/2751(95.1%)	100	855/916(93.3%)
**TbpB**	72	1009/1312(76.9%)	100	441/729(60.5%)
**PorB**	100	823/1053(78.2%)	100	236/350(67.4%)
**LbpA**	100	2720/2835(95.9%)	100	904/944(95.8%)
**LbpB**	96	1772/2202(80.5%)	100	520/768(67.7%)

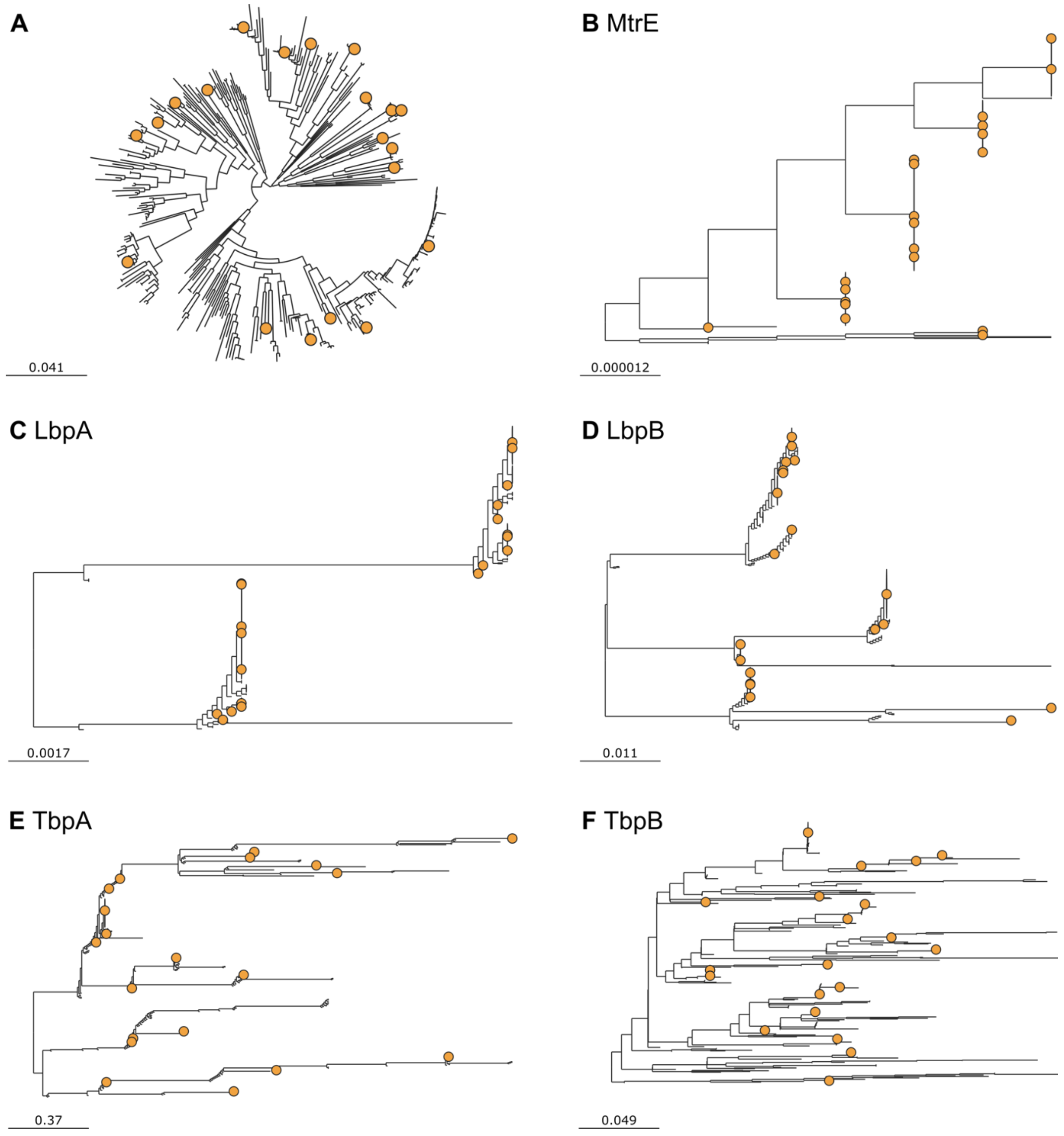

Supplementary Fig. 1 Mapping of the 20 selected *Neisseria gonorrhoeae* strains on the phylogenetic reconstruction of (A) the core genome of a set of 362 *N. gonorrhoeae* strains and the protein-based trees of the five key antigens, (B) MtrE, (C) LbpA, (D) LbpB, (E) TbpA and (F) TbpB, on this strain set. The scale bars represent, for A) the expected number of nucleotide substitutions per variable site along each branch, and for B-F) the expected number of amino acid substitutions per site along each branch.

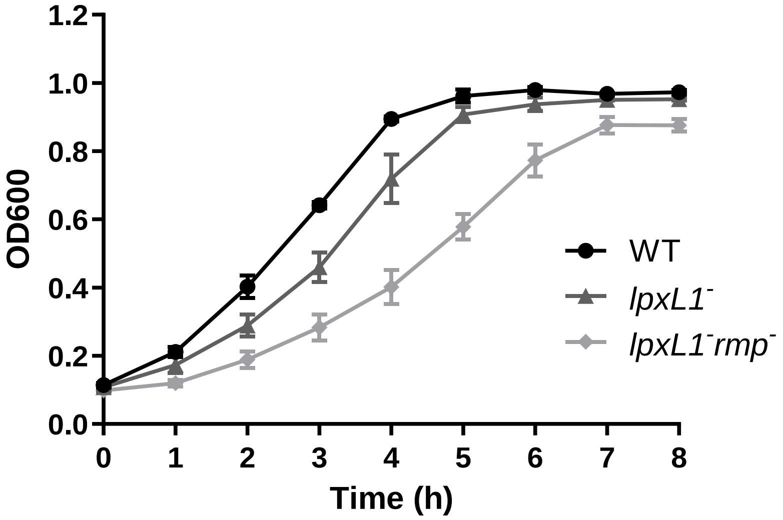

Supplementary Fig. 2 Growth curve of *N. gonorrhoeae* GC_0817560 wild-type and genetically modified strains. Liquid medium was inoculated to an initial OD_{600} of 0.1 from which sampled to measure the OD_{600} value were collected every hour. The experiment was repeated on three different days. Results represent mean±SD.

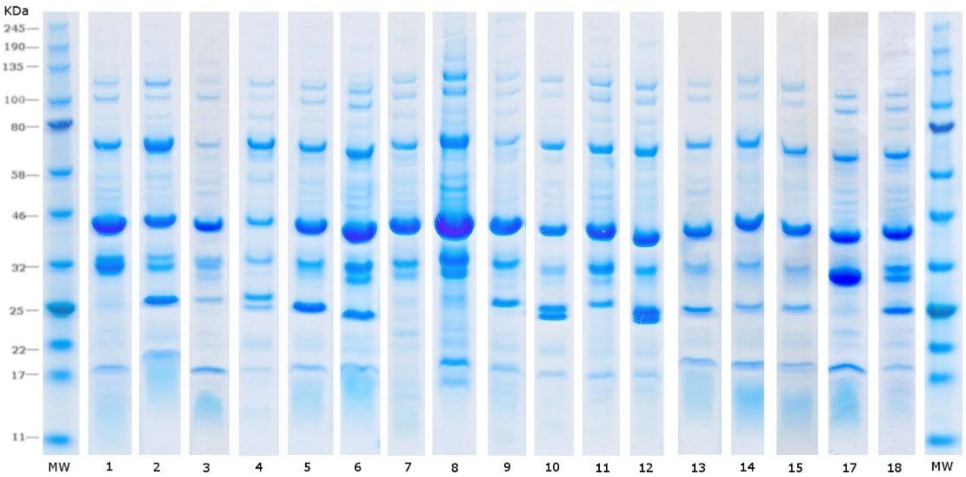

Supplementary Fig. 3 SDS-PAGE analysis of nOMV generated from 18 of the panel of 20 strains used for screening of vaccine production strain. Strain shown in table 1. MW molecular weight.

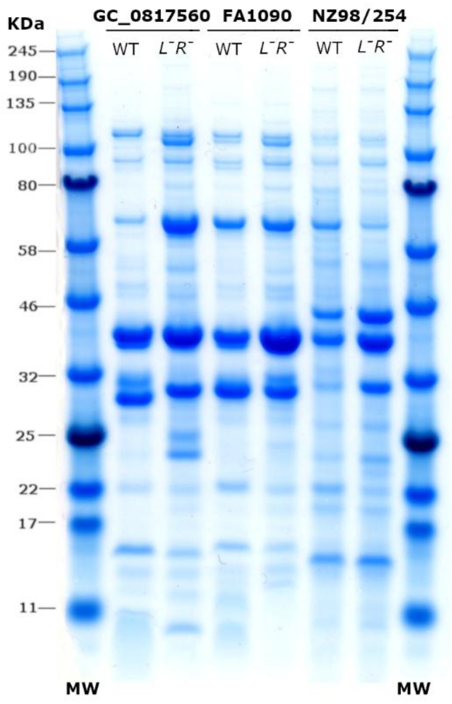

Supplementary Fig. 4 SDS-PAGE analysis of *Neisserial* nOMV derived from corresponding wild type (WT) and *lpxL1⁻rmp⁻* (*L⁻R⁻*) variants of *N. gonorrhoeae* GC_0817560 and FA1090, and *N. meningitidis* NZ98/254. 8 µg nOMV were loaded into each lane of a 4-12% polyacrylamide gradient gels using MES buffer. Gels were stained with Coomassie Blue.

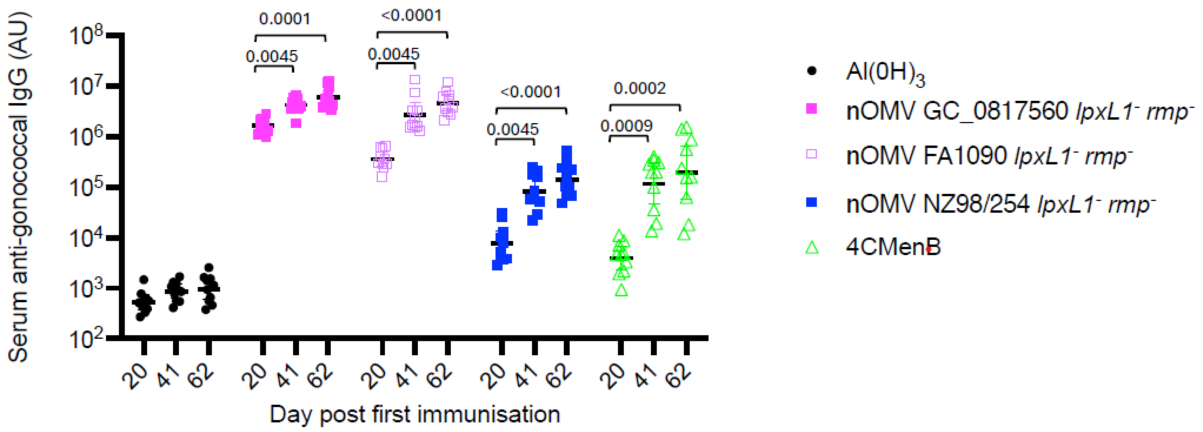

Supplementary Fig. 5 Time course of the serum anti-gonococcal IgG of mice immunised with nOMV GC_0817560 *lpxL1⁻rmp⁻*, nOMV FA1090 *lpxL1⁻rmp⁻*, nOMV NZ98/254 *lpxL1⁻rmp⁻* and 4CMenB. The same dataset as Fig.10 is graphed per day post immunisation to illustrate the time course. Six-week-old female BALB/c mice were immunized with the four vaccines formulated in $Al(OH)_3$ or $Al(OH)_3$ alone (adjuvant control, black circles) at 0, 3 and 6 weeks. Mice were bled 3 weeks after each immunisation (Days 20, 41 and 62). Serum anti-gonococcal IgG levels were determined by ELISA for 10 mice per group. ELISA coating substrate was GC_0817560 nOMV. Data within each group were analysed independently using Kruskal-Wallis and Dunn's correction for multiple comparison to characterises the changes in titre after two or three doses versus one dose, all significant results are indicated with the adjusted P value (n=10 mice per group).
